# Biomarkers That Correlate with Active Pulmonary Tuberculosis Treatment Response: a Systematic Review and Meta-analysis

**DOI:** 10.1128/jcm.01859-21

**Published:** 2022-02-16

**Authors:** Alexandra J. Zimmer, Federica Lainati, Nathaly Aguilera Vasquez, Carole Chedid, Sean McGrath, Andrea Benedetti, Emily MacLean, Morten Ruhwald, Claudia M. Denkinger, Mikashmi Kohli

**Affiliations:** a Department of Epidemiology, Biostatistics and Occupational Health, McGill University, Montreal, Canada; b McGill International TB Centre, Montreal, Canada; c Division of Clinical Tropical Medicine, Center of Infectious Diseases, Heidelberg University Hospital, Heidelberg, Germany; d Equipe Pathogenèse des Légionnelles, Centre International de Recherche en Infectiologie, INSERM U1111, Université Claude Bernard Lyon 1, CNRS UMR5308, École Normale Supérieure de Lyon, Lyon, France; e Medical and Scientific Department, Fondation Mérieux, Lyon, France; f Département de Biologie, Ecole Normale Supérieure de Lyon, Lyon, France; g Department of Biostatistics, Harvard T.H. Chan School of Public Health, Boston, USA; h FIND, Geneva, Switzerland; Boston Children's Hospital

**Keywords:** tuberculosis, treatment monitoring, biomarkers

## Abstract

Current WHO recommendations for monitoring treatment response in adult pulmonary tuberculosis (TB) are sputum smear microscopy and/or culture conversion at the end of the intensive phase of treatment. These methods either have suboptimal accuracy or a long turnaround time. There is a need to identify alternative biomarkers to monitor TB treatment response. We conducted a systematic review of active pulmonary TB treatment monitoring biomarkers. We screened 9,739 articles published between 1 January 2008 and 31 December 2020, of which 77 met the inclusion criteria. When studies quantitatively reported biomarker levels, we meta-analyzed the average fold change in biomarkers from pretreatment to week 8 of treatment. We also performed a meta-analysis pooling the fold change since the previous time point collected. A total of 81 biomarkers were identified from 77 studies. Overall, these studies exhibited extensive heterogeneity with regard to TB treatment monitoring study design and data reporting. Among the biomarkers identified, C-reactive protein (CRP), interleukin-6 (IL-6), interferon gamma-induced protein 10 (IP-10), and tumor necrosis factor alpha (TNF-α) had sufficient data to analyze fold changes. All four biomarker levels decreased during the first 8 weeks of treatment relative to baseline and relative to previous time points collected. Based on limited data available, CRP, IL-6, IP-10, and TNF-α have been identified as biomarkers that should be further explored in the context of TB treatment monitoring. The extensive heterogeneity in TB treatment monitoring study design and reporting is a major barrier to evaluating the performance of novel biomarkers and tools for this use case. Guidance for designing and reporting treatment monitoring studies is urgently needed.

## INTRODUCTION

In 2018, the global treatment success rate for people with drug-susceptible tuberculosis (TB) was 85% (1). Among the 7.0 million people reported to have received TB treatment in 2018, over 1 million individuals did not receive their treatment. Treatment success drops significantly among people with multidrug-resistant (MDR) TB and people living with HIV, with success rates of 57% and 76%, respectively (1). Continuous monitoring and early identification of people with TB who are at risk of poor treatment outcomes could reduce the number of people who do not complete treatment.

The World Health Organization (WHO) currently recommends sputum smear microscopy or culture conversion at the end of the intensive phase of treatment for monitoring treatment response in adults with pulmonary TB (2). However, these microbiology-based methods have limitations. Both smear microscopy and culture rely on sputum samples, which are not readily available in all populations (e.g., pediatric TB, people living with HIV, extrapulmonary TB) (3–5). Further, both methods are highly operator dependent (6). Smear microscopy is also not able to differentiate viable from nonviable TB, resulting in poor sensitivity and specificity for outcome prediction (7). For TB culture, the limited availability in primary care settings and the delay in time to results constrain its clinical use (8).

There is a clinical and public health need for new treatment monitoring biomarkers and assays that provide quick and accurate predictions of treatment outcomes. To meet the clinical needs for TB treatment monitoring, novel tests that detect biomarkers of interest would ideally be performed on noninvasive samples (e.g., blood, urine) and require limited laboratory expertise and infrastructure. Developments of tests based on host or pathogen biomarkers have previously been summarized in a narrative review article (9). A systematic assessment of these biomarkers is needed to identify those that might represent promising options to optimize treatment monitoring.

In this systematic review, we summarize, for the first time, a set of assays and biomarkers that correlate with TB treatment and, thus, may be of interest for TB treatment monitoring. We provide a summary of the biomarkers and assays identified as well as a more in-depth exploratory evaluation of the longitudinal change in levels of C-reactive protein (CRP), interferon gamma-induced protein 10 (IP-10), interleukin-6 (IL-6), and tumor necrosis factor alpha (TNF-α) during anti-TB treatment.

## MATERIALS AND METHODS

We conducted a systematic review of active pulmonary TB treatment monitoring biomarkers and assays that are commercial or have commercial potential. Study selection criteria for this review are illustrated in the PRISMA checklist (Table S1 in the supplemental material).

### Search strategy.

We searched six academic databases, including PubMed/MEDLINE, Embase, Web of Science, BIOSIS, Latin American and Caribbean Health Sciences (LILACS), and the Cochrane Database of Systematic Reviews. The full search strategy is presented in Table S2.

### Eligibility criteria.

Relevant studies published between 1 January 2008 and 31 December 2020 that were written in English were included. We included randomized clinical trials (RCTs), cohort studies, case-control studies, and cross-sectional studies that investigated the longitudinal change in biomarker levels during anti-TB treatment. We excluded case series, reviews, commentaries/editorials, case reports, mathematical modeling studies, economic analyses, and conference abstracts. We also excluded any study that did not perform reference standard testing at multiple time points throughout treatment and studies with a sample population of less than 10. Studies on children (age less than or equal to 15 years) were excluded given the difficulty of establishing a reference standard in this population. No restrictions were placed on the geographic area or the type of health system setting from where the participants were recruited. We extracted data for three categories of assays and biomarkers identified in consultation with the Foundation for Innovative New Diagnostics (FIND) technology scouting team, including (i) assays that are commercially available in a kit for research purposes, (ii) biomarkers not currently available in a commercial kit but are either under commercial development or have the potential to become commercial (e.g., transcriptomic signatures), and (iii) TB-specific biomarkers or commonly recognized laboratory procedures that are not necessarily commercialized (e.g., 16s rRNA molecular bacterial load assay [MBLA]). We did not include radiological methods or well-established assays such as sputum smear microscopy, culture, and nucleic acid amplification tests (e.g., GeneXpert, Hain). We included studies that used reference standards acceptable for treatment monitoring, which includes sequential measurements of Mycobacterium tuberculosis culture, Xpert MTB/RIF, smear microscopy, and/or clinical outcome. Measurements in comparison to the reference standard were included when at least one time point during treatment follow-up measured the reference standard.

### Screening and data extraction.

All publications identified from the search strategy were imported into the reference management database EndNote (version X9), after which duplicate citations were removed. Studies were screened by title and abstract by at least two reviewers (A.J.Z., C.C., N.A.V., and F.L.) before full-text screening. Prior to extraction, two authors (A.J.Z. and C.C.) piloted the data extraction forms independently on a random sample of five papers. An additional reviewer (M.K. and C.M.D.) screened studies for which the inclusion/exclusion criteria were not immediately clear. Two separate Google forms were piloted for data extraction, including (i) summary assessment to extract information relevant to the assay characteristics and study design, and (ii) quantitative assessment to extract biomarker levels (mean/median) and measures of spread (standard deviation, interquartile range) at each follow-up time point. All studies were extracted by at least two reviewers (A.J.Z., C.C., N.A.V., and F.L.). For the quantitative assessment, we only extracted data on biomarkers when quantitative changes in biomarker levels were reported by five or more studies (CRP, IP-10, IL-6, and TNF-α). For the biomarker levels and measures of spread, data were extracted directly from the texts or tables when available. If quantitative data were not available and authors did not respond to the request for data, the data (biomarker level and measure of spread) were extracted directly from available figures (dot plots, box plots, etc.) (10). These data were extracted in duplicate (N.A.V., F.L., and A.J.Z.), and one author (A.J.Z.) reviewed the extracted data and resolved any conflicts. When data were extracted from figures, one author (A.J.Z.) averaged the data across the two extractions.

### Assessment of quality and risk of bias.

We evaluated the quality and risk of bias of all included studies for the four domains of the Quality Assessment of Diagnostic Accuracy Score 2 (QUADAS-2), including patient selection, index test, reference standard, and flow and timing. Each domain was evaluated using a set of QUADAS-2 guiding questions (Table S3). Items were scored as “high concern,” “low concern,” or “unclear concern.” The overall risk of bias (Fig. S4) was evaluated as “high risk” for studies with more than one area of high concern, “intermediate risk” for all studies that included one area of high concern, “low risk” for all studies with two or more areas of low concern and no high risk, and “unclear risk” for all studies with three or more areas of unclear concern and no high risk. No commercialized assay for the specific use case of treatment monitoring was identified. Given this, no validated cutoff exists for biomarkers with a quantitative output for monitoring TB response. Thus, cutoffs were not part of the QUADAS-2 assessment for the included studies.

### Data analysis.

We investigated how biomarker levels changed over time. When biomarkers had five or more studies that numerically or graphically presented the measures of central tendency and measures of spread at different follow-up time points (10), we evaluated the fold change in biomarker levels relative to the previous time point collected. For this analysis, we did not include studies that used TB-antigen stimulated samples.

We first standardized the data extracted into sample mean and standard deviation values. Specifically, we applied the Box-Cox (BC) method proposed by McGrath et al. to estimate the sample mean and standard deviation from studies that reported the median and first and third quartiles (11, 12). In one study with highly skewed data at some time points (CRP from Ferrian et al.), the BC method produced estimates that were biologically implausible. For this study, we estimated the sample means and standard deviations by maximum likelihood with several candidate models (normal, log-normal, gamma, Weibull) and selected the model with the largest likelihood.

The fold change at each follow-up time was calculated as the difference between the current and previously recorded value divided by the previously recorded value as follows:

$${\text{fold change}}_{t}=\frac{{\text{mean biomarker level}}_{t}-{\text{ mean biomarker level}}_{t-1}}{{\text{mean biomarker level}}_{t-1}}$$

Fold changes were plotted separately for each study (Fig. S7). When studies reported the change in biomarker level across different groups of patients (e.g., fast responders, slow responders), we pooled the results to examine the average changes in biomarker level across patients that responded to treatment. Fast responders were generally defined as individuals who experienced culture conversion before 8 to 12 weeks of treatment, while individuals who experienced culture conversion beyond 8 to 12 weeks of treatment were defined as slow responders.

To characterize how biomarker levels change with respect to treatment, we performed two meta-analyses, (i) a meta-analysis of the fold change in biomarker levels between baseline and 8 weeks of treatment for studies that reported biomarker levels at 8 weeks (the end of the intensive phase of treatment), and (ii) a meta-analysis pooling fold change since previous time point using a random intercept model. Both analyses used the metafor package for R (version 4.0.6) at the study level (13). For estimated fold change of each biomarker, 95% confidence intervals were also calculated to assess the statistical significance for each biomarker. As is common in longitudinal meta-analyses, the primary studies did not report data on the correlation between the effect estimates at the different follow-up times. For each biomarker, we constructed approximate covariance matrices of the study-specific effect estimates by assuming that the correlation between all pairs of mean biomarker values in a given study was the same value ρ (14). We used the correlation parameter, ρ = 0.5, in the primary analysis and used ρ values of 0, 0.25, and 0.75 in sensitivity analyses (Table S8). We also included a sensitivity analysis for the random intercept CRP model, including and excluding Ferrian et al (Table S9). The list of studies included in the week 8 meta-analysis and the fold change meta-analysis can be found in Table S6. The code for all analyses is publicly available on GitHub (https://github.com/stmcg/tmsr).

## RESULTS

### Search results.

After removing duplicate records, 8,795 publications were screened (title and abstract). Among these, 441 were identified for full-text review, of which 77 were included in the review for the summary (qualitative) assessment (15–91). Nineteen of the records were included in the detailed (quantitative) assessment, including the meta-analyses for the biomarkers CRP, IP-10, IL-6, and TNF-α.

**FIG 1:**
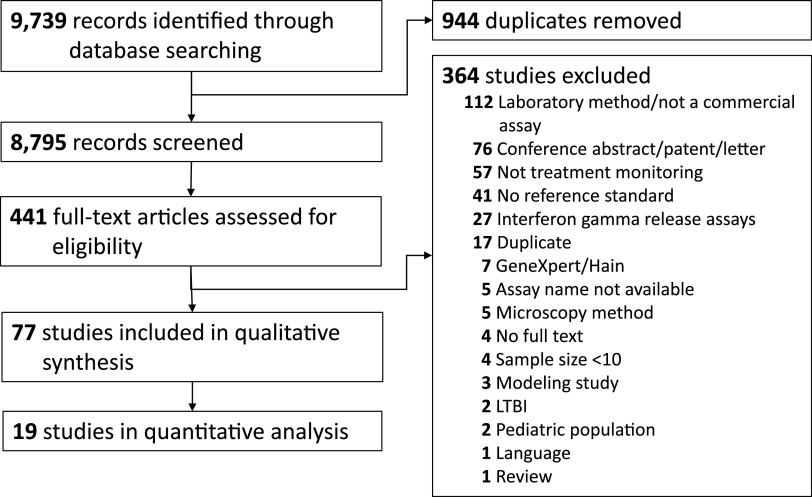
PRISMA flow diagram for literature search and paper selection.

Out of the 441 records that underwent full-text screening, 112 were excluded because the assays did not align with any of the three predefined assays of interest (with regard to commercialization and relevance to TB treatment monitoring). The majority of these were in-house laboratory methods (Fig. 1). Fifty-seven of the studies excluded were not treatment monitoring studies, while 41 did not utilize reference standards during follow-up and/or by the end of therapy. Studies that examined well-established diagnostics, such as interferon gamma release assays (IGRAs; *n* = 27), GeneXpert/Hain (*n* = 7), and microscopy methods (*n* = 5), were excluded, as prior systematic reviews have already characterized the treatment monitoring capabilities of these assays (7, 92–94).

### Characteristics of included studies.

General study demographics and characteristics are summarized in Table 1. Most of the studies were limited to the discovery phase and were conducted in single-center studies in a single country (96%). All but two examined patients from medium-high TB burden countries (94%). Participants with drug resistance (including multidrug resistance) at baseline were included in 26% of studies. More than half of the studies did not indicate whether participants had a history of prior TB, and 88% of studies did not indicate whether participants had previously received the bacillus Calmette-Guérin vaccine. Finally, about one-quarter of studies included people living with HIV.

**TABLE 1:** Study characteristics of the 77 studies included in the qualitative synthesis

Study characteristic^*c*^	Value (no. [%]) (*n* = 77)
TB burden of country of enrollment^*a*^	
Low (<10 cases per 100,000 population per yr)	2 (2.60)
Middle (11 to 40 cases per 100,000 population per yr)	8 (10.39)
High (>40 cases per 100,000 population per yr and/or WHO list of 30 highest burden countries)	64 (83.12)
Multisite^*b*^	3 (3.90)
	
Persons with drug resistance at baseline included	
Yes	20 (25.97)
No	20 (25.97)
Unclear/not reported	37 (48.05)
	
Persons with a history of prior TB included	
Yes	17 (22.08)
No	21 (27.27)
Unclear/not reported	39 (50.65)
	
Persons with BCG vaccination included	
Yes	9 (11.69)
Unclear/Not reported	68 (88.31)
	
Persons living with HIV included	
Yes	19 (24.68)
No	45 (58.44)
Unclear/not reported	13 (16.88)

a Based on 2019 data.

b Three studies recruited participants from different countries with different TB burden status.

c TB, tuberculosis; WHO, World Health Organization; BCG, *Bacillus* Calmette-Guérin.

### Quality and risk of bias assessment (QUADAS-2).

**FIG 2:**
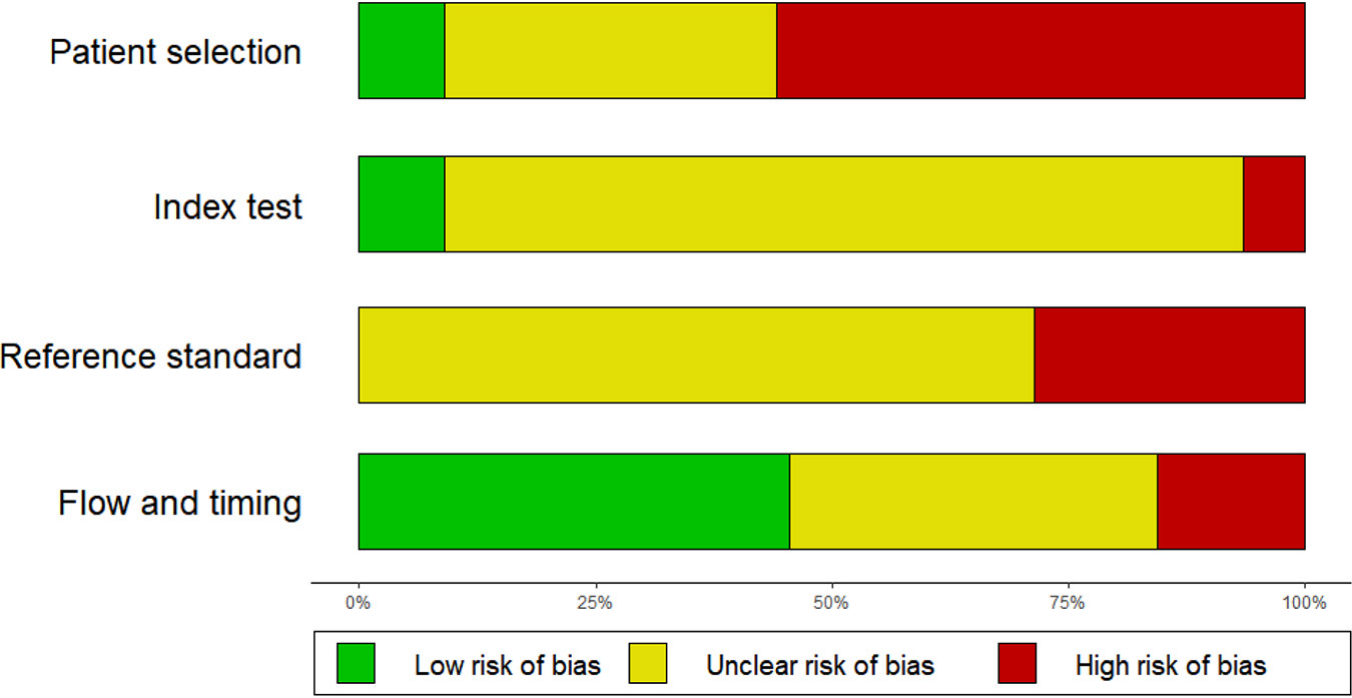
Summary of the QUADAS-2 risk of bias assessment.

When considering the four main categories of the QUADAS-2 quality and risk of bias assessment tool, “patient selection,” “index test,” “reference standard,” and “flow and timing,” only three studies (4%) had an overall low risk of bias (Fig. S4 in the supplemental material). The QUADAS-2 assessments are summarized in Fig. 2. Specifically, the risk of bias for patient selection was high for studies that used a case-control study design. Many studies excluded smear-negative participants, which also introduced bias in the patient selection strategy. Most studies did not report whether the reference standard was blinded while interpreting the results of the index. Regarding treatment monitoring reference standards, all studies that used culture as a reference standard received an “unclear risk of bias” since the accuracy of culture for this use case is not 100%. Studies that used smear microscopy received a “high risk of bias.” Finally, the flow and timing of the study were generally “low risk of bias,” as the majority of samples were either frozen or processed immediately. For some studies, the loss to follow-up throughout the treatment monitoring period resulted in a high risk of bias.

### Summary assessment of treatment monitoring biomarkers.

Across all studies, 81 different biomarkers were identified (Table S5). Forty-nine biomarkers were evaluated in just one study. Most of the biomarkers were host-response markers, with the exception of lipoarabinomannan (LAM) in urine, sputum, and plasma (30, 34, 48, 86), 16s rRNA in sputum (39, 41, 42, 75, 87), 85B mRNA in sputum (19, 60, 64, 81), and IS6110 insertion element in sputum (60). Among the host-response biomarkers, most biomarkers were cytokine proteins measured in blood, both proinflammatory (e.g., IL-1, IL-6, and TNF-α) and anti-inflammatory (e.g., IL-4 and IL-10), which were most commonly analyzed using plasma or serum samples on commercially available research enzyme-linked immunosorbent assay (ELISA) kits. Several chemokines were also investigated, including interferon-inducible T cell alpha chemoattractant (I-TAC), monokine induced by interferon gamma (MIG), and IP-10 (also known as CXCL11, CXCL9, and CXCL10, respectively) (26, 27, 56). Of all biomarkers, IP-10 was the most frequently analyzed biomarker for treatment monitoring, with 11 studies investigating longitudinal changes in marker level (23, 27, 29, 33, 36, 43, 45, 47, 51, 59, 72, 90). Several blood-based transcriptomic and gene expression signatures were examined as treatment monitoring markers, including the parsimonious 3-gene Sweeney3 signature (35, 85), the RISK6 signature (69), and the RISK11 signature (28). Two studies explored changes in breath-based markers such as fractional exhaled nitric oxide (FeNO) and volatile organic compounds (VOCs) (71, 88), while another study by Lee et al. characterized changes in cough frequency throughout treatment (55). Table 2 summarizes the biomarkers that were included in the detailed quantitative assessment, as well as the transcriptomic signatures.

**TABLE 2:** Assays and study characteristics for treatment monitoring biomarkers of interest^*a*^

Biomarker	Author (yr)	Reference no.	Assay name (manufacturer)	County	Follow-up times	Sample(s) (state)	Reference(s) used
C-reactive protein (CRP)	Almeida (2009)	15	Roche CRPLX kit on the Roche modular analyzer (Roche)	Brazil	0, W1, W3, W5, W8	Plasma (frozen)	Solid culture (NR), clinical outcome, smear microscopy (NR)
	Djoba Siawaya (2008)	78	CRP ELISA kit (Bender MedSystems)	South Africa	0, W1, W5, W13, W26	Serum (frozen)	Chest X-ray, liquid culture (BACTEC)
	Ferrian (2017)	33	CRP on a multiplex (ProcartaPlex human kits; eBioscience) read on a luminometer (Bio-Plex 200; BioRad)	South Africa	0, M2, M4, M6	Plasma (frozen)	Smear microscopy (NR), liquid culture (MGIT 960), line probe assay (Hain)
	Francisco (2017)	35	Quantikine ELISA (R&D Systems Inc., Minneapolis, MN, USA)	China	0, M4, M6, M7	Whole blood (NR)	Clinical outcome
	Jayakumar (2015)	45	ichroma CRP point-of-care reader (BodiTech Med Inc.)	Uganda	0, W8, W20	Serum (frozen)	Solid culture (NR), liquid culture (MGIT 960)
	Khalil (2020)	49	NycoCard CRP reader II (Abbott)	Egypt	M1, M2, M3	Whole blood (NR), plasma (NR), serum (NR)	Solid culture (LJ), clinical outcome, smear microscopy (ZN), GeneXpert (MTB/RIF)
	Mendy (2016)	61	CRP ELISA kit (Immunology Consultants Laboratory)	Gambia	0, M2, M6	Plasma (frozen)	Liquid culture (MGIT), smear microscopy (ZN), chest X-ray
	Mesquita (2016)	62	CRP ELISA kit (Ebioscience)	Brazil	0, D60	Serum (frozen)	Culture (NR), chest X-ray
	Miranda (2017)	63	CRP ELISA kit (Ebioscience)	Brazil	0, D30, D60	Serum (frozen)	Solid culture (LJ)
	Moraes (2014)	65	CRP BNII nephelometer (Dade Behring)	Brazil	0, D30, D60	Serum (fresh)	Solid culture (LJ), clinical outcome, smear microscopy (ZN)
	Sigal (2017)	80	CRP V-Plex human vascular injury panel 2 (Meso Scale Diagnostics)	North America, Spain, South Africa, Uganda	0, W8, W12	Serum (frozen)	Solid culture (LJ), liquid culture (MGIT 960), clinical outcome, chest X-ray
IL-6	Chowdhury (2014)	25	IL-6 ELISA kit (RayBiotech)	India	0, M2, M4, M6	Serum (NR)	Smear microscopy (ZN), chest X-ray
	Djoba Siawaya (2009)	29	IL-6 Lincoplex human cytokine 29-plex assays (Millipore)	South Africa	0, W1, W5, W13, W26	Plasma (frozen)	Liquid culture (BACTEC 460T), smear microscopy (NR)
	Feng (2020)	32	IL-6 Quantikine ELISA kit (R&D Systems)	Taiwan	0, W8	Peripheral blood mononuclear cells (frozen)	Culture (NR), smear microscopy (NR)
	Luo (2018)	57	IL-6 ELISA kits (Siemens Healthcare Diagnostics Products Ltd., Llanberis, Gwynedd, UK)	China	0, M2	Serum (fresh)	Smear microscopy (FM)
	Mvungi (2019)	66	IL-6 multiplex assay (human premixed multianalyte kit; catalog no. LXSAHM) on the Luminex 200 system	Tanzania	0, M2	Plasma (frozen) prepared using QuantiFERON-TB Gold Plus	Clinical outcome
	Riou (2012)	72	IL-6 multiplex bead array (bulletin no. 10014905; Bio-Rad) on a luminometer (Luminex)	South Africa	0, W2, W4, W8, W12, W26, W52, W78	Plasma (frozen)	Solid culture (LJ), liquid culture (MGIT), smear microscopy (FM)
	Sigal (2017)	80	IL-6 V-Plex human proinflammatory panel 1 (Meso Scale Diagnostics)	North America, Spain, South Africa, Uganda	0, W8, W12	Serum (frozen)	Solid culture (LJ), liquid culture (MGIT 960), clinical outcome, chest X-ray
IP-10	Chen (2011)	23	IP-10 ELISA (R&D Systems)	Taiwan	0, M2, M6	Serum (NR)	Solid culture (LJ), liquid culture (MGIT), smear microscopy (ZN), chest X-ray
	Chung (2016)	27	IP-10 ELISA (R&D systems, Minneapolis, MN)	South Korea	0, M2	Serum (frozen)	Culture (NR), smear microscopy (NR), clinical outcome, chest X-ray
	Djoba Siawaya (2009)	29	IP-10 Lincoplex human cytokine 29-plex assays (Millipore)	South Africa	0, W1, W5, W13, W26	Plasma (frozen)	Liquid culture (BACTEC 460T), smear microscopy (NR)
	Ferrian (2017)	33	IP-10 on a multiplex (ProcartaPlex human kits; eBioscience) read on a luminometer (Bio-Plex 200; BioRad)	South Africa	0, M2, M4, M6	Plasma (frozen)	Smear microscopy (NR), liquid culture (MGIT 960)
	Francisco (2017)	35	ELISA kit (RayBiotech, Inc.)	China	0, M4, M6, M7	Whole blood (NR)	Clinical outcome
	Garcia-Basteiro (2017)	36	IP-10 ELISA kit (Becton Dickinson and Company)	Mozambique	0, D7, D60	Serum (frozen)	Smear microscopy (ZN), GeneXpert (MTB/RIF), liquid culture (MGIT 960)
	Hong (2014)	43	IP-10 ELISA kit (R&D Systems)	South Korea	0/within W2, after M6-M9	Serum (NR)	Culture (NR), chest X-ray, CT scan
	Jayakumar (2015)	45	IP-10 ELISA kit (R&D Systems)	Uganda	0, W8, W20	Serum (frozen)	Solid culture (NR), liquid culture (MGIT 960)
	Kabeer (2011)	47	IP-10 ELISA kit (R&D Systems) in response to QFT-IT and RD1	India	0, M6	Plasma (fresh)	Solid culture (LJ), liquid culture (BacT)
	Kim (2018)	51	IP-10 ELISA kit (R&D Systems, Minneapolis, MN, USA)	South Korea	0, M6, M12	Urine (NR), Serum (NR)	Culture (NR), chest X-ray
	Matsushita (2015)	59	IP-10 27-plex assay on the Bio-Plex suspension array system (Bio-Rad)	Vietnam	0, M2, M7	Plasma (frozen)	Smear microscopy (NR), chest X-ray
	Riou (2012)	72	IP-10 multiplex bead array (bulletin no. 10014905; Bio-Rad) on a luminometer (Luminex)	South Africa	0, W2, W4, W8, W12, W26, W52, W78	Plasma (frozen)	Solid culture (LJ), liquid culture (MGIT), smear microscopy (FM)
	Zhu (2015)	90	IP-10 ELISA kit (eBioscience)	China	0, W2-8	Plasma (frozen)	Smear microscopy (NR)
TNF-α	Chowdhury (2014)	25	TNF-α high-sensitivity human ELISA set (ImmunoTools)	India	0, M2, M4, M6	Serum (NR)	Smear microscopy (ZN), chest X-ray
	Djoba Siawaya (2009)	29	TNF-α Lincoplex human cytokine 29-plex assays (Millipore)	South Africa	0, W1, W5, W13, W26	Plasma (frozen)	Liquid culture (BACTEC 460T), smear microscopy (NR)
	Luo (2018)	57	TNF-α ELISA kit (Siemens Healthcare Diagnostics)	China	0, M2	Serum (fresh)	Smear microscopy (FM)
	Mvungi (2019)	66	TNF-α multiplex assay (Human premixed multianalyte kit; catalog no. LXSAHM) on the Luminex 200 system	Tanzania	0, M2	Plasma (frozen) using QTF-TB Gold Plus	Clinical outcome
	Nie (2020)	67	TNF-α ELISA kit (BioLegend)	China	0, M1-2, M6	Serum (frozen)	Culture (NR), smear microscopy (NR), chest computed tomography
	Riou (2012)	72	TNF-α multiplex bead array (bulletin no. 10014905; Bio-Rad) on a luminometer (Luminex)	South Africa	0, W2, W4, W8, W12, W26, W52, W78	Plasma (frozen)	Solid culture (LJ), liquid culture (MGIT), smear microscopy (FM)
	Zhu (2015)	90	TNF-α ELISA kit (eBioscience)	China	0, W2-8	Plasma (frozen)	Smear microscopy (NR)
Transcriptomic/gene signatures	Bloom (2012)	21	664-Transcript signature, 320-transcript signature	South Africa	0, W2, M2, M6, M12	Whole blood (frozen)	Clinical outcome, chest X-ray
	Darboe (2019)	28	RISK11 signature	South Africa	0, M2, M6, M8, M14	Whole blood (NR)	Culture (NR)
	Francisco (2017)	35	3-gene signature (*GBP5*, *DUSP3*, and *KLF2*)	China	0, M4, M6, M7	Whole blood (NR)	Clinical outcome
	Gebremicael (2019)	38	105 genes expression profiling by dual-color reverse-transcription multiplex ligation-dependent probe amplification (dcRT-MLPA) platform	Ethiopia	0, M6, M18	Whole blood (NR)	Smear microscopy (ZN)
	Penn-Nicholson (2020)	69	RISK6 signature	South Africa	0, M2, treatment completion, 6 to 8 mo posttreatment	Whole blood (frozen)	Culture (NR), smear microscopy (NR), GeneXpert (MTB/RIF)
	Sivakumaran (2020)	82	198-gene set profiled using dc-RT MLPA platform	India	0, M1, M2, M6	Whole blood (frozen)	Liquid culture (MGIT), smear microscopy (FM), clinical outcome
	Warsinske (2018)	85	3-gene signature (*GBP5*, *DUSP3*, and *KLF2*)	South Africa	0, W1, W4, W24	Whole blood (NR)	Liquid culture (MGIT), PET-CT

a D, day; W, week; M, month; NR, not reported; MGIT, mycobacteria growth indicator tube; LJ, Löwenstein-Jensen; ZN, Ziehl-Neelsen; FM, fluorescent microscopy; PET-CT, positron emission tomography-computed tomography; QFT-IT, QuantiFERON-TB Gold In-Tube test; dcRT-MLPA, dual-color reverse-transcription multiplex ligation-dependent probe amplification.

### Detailed quantitative assessment of treatment monitoring biomarkers.

For biomarkers where there were five or more studies that numerically or graphically presented the measures of central tendency and measures of spread at different follow-up time points, we further characterized the week 8 fold change and fold changes with respect to previously reported time points. Results of the meta-analysis found that CRP, IP-10, IL-6, and TNF-α (Table 3) decreased by week 8 of treatment compared to baseline (week 0). CRP experienced the greatest week 8 fold change of −76.1% (95% confidence interval [CI], −89.4% to −62.9%) while TNF-α had the smallest fold change of −10.3 (95% CI, −24.7% to −4.2%). Both IL-6 and IP-10 experienced fold changes of −24.7% (95% CI, −50.7% to 1.3%) and −38.2% (95% CI, −61.3% to −15.0%), respectively, though the confidence interval for IL-6 crossed the null.

**TABLE 3:** Pooled week 8 fold change and fold change since previously recorded time point of CRP, IL-6, IP-10, and TNF-α among people with TB on therapy^*d*^

Biomarker	Data for baseline to week 8			Data since previously recorded time point		
	No. of studies^*a*^	No. of participants	Avg fold change (% [95% CI])	No. of Studies	No. of participants^*b*^	Avg fold change (% [95% CI])
CRP	5	275	−76.1 (−89.4 to −62.9)	7	447^*c*^	−53.9 (−70.2 to −37.5)
IL-6	4	522	−24.7 (−50.7 to 1.3)	5	558	−31.0 (−59.5 to −2.5)
IP-10	4	154	−38.2 (−61.3 to −15.0)	9	430	−36.2 (−49.0 to −23.4)
TNF-α	4	497	−10.3 (−24.7 to −4.2)	6	517^*c*^	−17.7 (−31.3 to −4.0)

a Only includes studies that collected data at week 8 (Table S6 in the supplemental material).

b At enrollment.

c Number of participants in Zhu et al. (90) was not specified.

d CRP, C-reactive protein; IL-6, interleukin-6; IP-10, interferon gamma-induced protein 10; TNF-α, tumor necrosis factor alpha; CI, confidence interval.

We further investigated the fold change of these four biomarkers with respect to the previously recorded time point (Fig. S7). The results of our meta-analysis found that there was a statistically significant decrease in levels of all four biomarkers with respect to the previous recorded value. Results of this meta-analysis complement the findings of the week 8 meta-analysis. CRP had the largest average change in biomarker level of −53.9% (95% CI, −70.2% to −37.5%) relative to the previously recorded time point. TNF-α had the smallest average fold change of −17.7% (95% CI, −31.3% to −4.0%) relative to previously recorded time points. Both IL-6 and IP-10 levels experienced a similar average changes of −31.0% (95% CI, −59.5% to −2.5%) and −36.2% (95% CI, −49.0% to −23.5%). For all biomarkers, confidence intervals were narrow, and the same conclusions were obtained in the sensitivity analyses where we varied the assumed correlation value (Table S8).

## DISCUSSION

In this systematic review, we examined the current landscape of assays used to evaluate changes in biomarker levels with respect to TB treatment. Host inflammation markers, including CRP, IP-10, IL-6, and TNF-α, were some of the most commonly evaluated biomarkers for TB treatment response. CRP and IP-10 have been particularly well characterized as a biomarker for TB screening and diagnosis in other studies (95, 96). However, we identified 81 different biomarkers that were evaluated in the context of TB treatment monitoring. Thus, while these four biomarkers appear to be promising given our exploratory quantitative analyses and should be further investigated, research into other, more novel biomarkers for TB treatment monitoring remains important.

From our quantitative analyses, we observed that, for the average fold change between baseline and week 8 of treatment, CRP, IP-10, and TNF-α had a statistically significant decrease. This analysis informs us of the average magnitude of the decrease in biomarker level between baseline and the end of the intensive phase of treatment. The results of our meta-analysis found that, on average, all four biomarkers decreased with respect to previously recorded time points. Out of the four biomarkers analyzed, CRP had the largest absolute week 8 fold change value of −76.1% (95% CI, −89.4% to −62.9%) and fold change relative to previous recorded time points of −53.9% (95% CI, −70.2% to −37.5%). This early response during the intensive phase of TB treatment and continued fold change throughout treatment may help with clinical decision-making by identifying people who respond favorably to treatment, though further analyses are needed to characterize how this fold change differs between people who respond to treatment and people who do not respond to treatment or are lost to follow-up. In addition, further investigations are required, as most of the included studies recruited a narrow patient spectrum, making the generalizability of the results a challenge.

Since these host inflammation markers are usually obtained from blood, serum, and/or plasma samples, they provide an advantage over traditional sputum-based methods such as microscopy and culture. However, as the detected changes were small, obtaining accurate readings in a timely, near-patient manner will be difficult. Nevertheless, the changes were statistically significant, which may suggest they may have potential to support clinical decision-making for TB treatment monitoring.

Among the host noninflammatory biomarkers, blood-based transcriptomic and gene expression signatures have gained significant momentum for TB diagnostics and treatment monitoring. The ability to detect the up- or downregulation of specific genes may allow for simpler and earlier identification of people who respond both favorably and unfavorably to treatment. As these signatures become increasingly parsimonious, their potential for commercialization into assays that run on standard PCR machines increases. Cepheid (USA) recently developed a prototype cartridge assay that runs on the GeneXpert platform for the Sweeney3 (3-gene signature) called the Xpert MTB Host Response or Xpert-MTB-HR-Prototype. A recent study performed a preliminary investigation on the performance of the Xpert-MTB-HR-Prototype as a treatment monitoring tool among 31 patients with pulmonary TB and found that the signature correlated with treatment progression (97). So far, each of the transcriptomic signatures identified in this review has only been evaluated in a limited number of cohorts, preventing us from meta-analyzing the fold change of these markers throughout treatment. Additional well-conducted studies are needed to quantitatively evaluate the performance of these signatures for treatment monitoring. Promising gene signatures that should be evaluated further in the context of TB treatment monitoring include Sweeney3, RISK6, and RISK11 (28, 35, 69, 85).

There are several limitations associated with this study. First, because not all studies reported the exact biomarker levels for CRP, IP-10, IL-6, and TNF-α, some of the data had to be extracted from figures, which may have introduced measurement error in the quantitative analyses. We attempted to mitigate this bias by extracting the estimates in duplicate. Further, a recent study by Mierden et al. found that the error from empirical evaluation of data from figures is often inconsequential and that “data extraction from graphs is a good method to harvest data if it is not provided in the text or tables” (10). Second, most, if not all, biomarkers were evaluated using different assays in each study. For example, across the 10 studies that evaluated CRP, 9 different assays were used, including 3 different ELISA kits (61–63, 78), 2 multiplex kits (33, 80), 1 nephelometer (65), 2 assays on point-of-care modules (one by Abbott, the other by BodiTech Med) (45, 49), and 1 assay on the Roche modular analyzer (15). This heterogeneity and consequent variability in assay performances could not be accounted for in the analyses. Third, we compared studies with different patient characteristics (e.g., different HIV status levels, fast versus slow responders, different proportion of drug-resistant or multidrug-resistant TB, etc.). Because the majority of studies did not disaggregate biomarker-level data by patient characteristic or treatment regimen, we were unable to perform subgroup analyses comparing how the fold change in biomarkers differed across populations. Fourth, the results of the meta-analysis for the fold change relative to previously recorded time points is entirely dependent on the data collected in the included studies. Given the high risk of bias and extensive heterogeneity across the studies, the quantitative fold change results are exploratory and limited in interpretation outside the context of our systematic review. Nevertheless, these preliminary data may help inform future studies to investigate these biomarkers in a more rigorous and standardized manner for TB treatment monitoring. Finally, this study does not explain the biological reason for the change in marker levels over time, which is essential for understanding the treatment monitoring potential of the biomarker. Further evaluations are needed to understand whether such changes in biomarker levels directly inform us that the treatment is effective. Additionally, studies are needed to characterize how biomarkers would respond to partially effective and ineffective regimens.

It is important to highlight that the overall quality of studies evaluated was poor, suggesting an overall high risk of bias with respect to the reference standard, index test, and patient selection of QUADAS-2 domains. What is most concerning, however, is the extensive heterogeneity in the study design and data reporting strategies across TB treatment monitoring studies. This heterogeneity limited our ability to properly evaluate the performance of the biomarkers and assays. Lack of uniform follow-up time points and reporting strategies, inconsistent definitions of treatment success versus treatment failure, and variability in the type and timing of reference standards were some of the key issues that complicated the evaluation of biomarkers and assays. Treatment monitoring of active pulmonary TB is an essential part of TB care, and yet, there is very little guidance on best practices for researchers on how to design studies and evaluate the accuracy and characteristics of treatment monitoring biomarkers and assays, and even less guidance for clinicians to use these different biomarkers to inform TB treatment progression among patients. Our systematic review and meta-analysis highlights that while TB treatment monitoring is an active area of research, additional work is needed to formulate appropriate study guidelines, gain clear consensus regarding stakeholder needs through WHO-endorsed TB target product profiles (TPPs), and inform clinical decision-making (98).
