# Supplementary material for: Biomarkers That Correlate with Active Pulmonary Tuberculosis Treatment Response: a Systematic Review and Meta-analysis
Source: J Clin Microbiol. 2022 Feb 16;60(2):e01859-21. doi: 10.1128/jcm.01859-21 (PMC8849205; doi:10.1128/jcm.01859-21)
Supplement: Supplemental file 1 — Table S1, Text S2, Table S3, Fig. S4, Tables S5 and S6, Fig. S7, and Tables S8 to S10. Download jcm.01859-21-s0001.pdf, PDF file, 0.8 MB [file jcm.01859-21-s0001.pdf]

Supplementary file

Table of contents

Table S1: PRISMA Checklist

S2: Search strategy

Table S3: QUADS-2 guiding questions

Figure S4: Study-specific QUADAS-2 scores

Table S5: Complete list of studies and biomarkers included in qualitative analysis

Table S6: Full list of studies and references included in the quantitative fold-change analyses

Figure S7: Fold-changes plots for individual studies of CRP, IP-10, IL-6 and TNF-α

Table S8: Sensitivity analysis for the weekly fold-change meta-regression with varying correlation coefficient (ρ) parameter

Table S9: Meta-analysis of outcome is the fold-change from previous recorded timepoint sensitivity analysis for CRP (excluding Ferrian et al.)

Table S10: Meta-regression where the outcome is the fold-change from baseline and the predictor is treatment duration.

Table S1: PRISMA Checklist

Section/topic	#	Checklist item	Reported on page #
**TITLE**			
Title	1	Identify the report as a systematic review, meta-analysis, or both.	p.1
**ABSTRACT**			
Structured summary	2	Provide a structured summary including, as applicable: background; objectives; data sources; study eligibility criteria, participants, and interventions; study appraisal and synthesis methods; results; limitations; conclusions and implications of key findings; systematic review registration number.	p.2
**INTRODUCTION**			
Rationale	3	Describe the rationale for the review in the context of what is already known.	p.4
Objectives	4	Provide an explicit statement of questions being addressed with reference to participants, interventions, comparisons, outcomes, and study design (PICOS).	p.5
**METHODS**			
Protocol and registration	5	Indicate if a review protocol exists, if and where it can be accessed (e.g., Web address), and, if available, provide registration information including registration number.	
Eligibility criteria	6	Specify study characteristics (e.g., PICOS, length of follow-up) and report characteristics (e.g., years considered, language, publication status) used as criteria for eligibility, giving rationale.	p.6
Information sources	7	Describe all information sources (e.g., databases with dates of coverage, contact with study authors to identify additional studies) in the search and date last searched.	p.5
Search	8	Present full electronic search strategy for at least one database, including any limits used, such that it could be repeated.	Supplement S2
Study selection	9	State the process for selecting studies (i.e., screening, eligibility, included in systematic review, and, if applicable, included in the meta-analysis).	p.6
Data collection process	10	Describe method of data extraction from reports (e.g., piloted forms, independently, in duplicate) and any processes for obtaining and confirming data from investigators.	p.6-7

Data items	11	List and define all variables for which data were sought (e.g., PICOS, funding sources) and any assumptions and simplifications made.	p.7
Risk of bias in individual studies	12	Describe methods used for assessing risk of bias of individual studies (including specification of whether this was done at the study or outcome level), and how this information is to be used in any data synthesis.	p.7-8
Summary measures	13	State the principal summary measures (e.g., risk ratio, difference in means).	p.8-9
Synthesis of results	14	Describe the methods of handling data and combining results of studies, if done, including measures of consistency (e.g., I^2) for each meta-analysis.	p.9
Risk of bias across studies	15	Specify any assessment of risk of bias that may affect the cumulative evidence (e.g., publication bias, selective reporting within studies).	p.7-8
Additional analyses	16	Describe methods of additional analyses (e.g., sensitivity or subgroup analyses, meta-regression), if done, indicating which were pre-specified.	p.9-10
**RESULTS**			
Study selection	17	Give numbers of studies screened, assessed for eligibility, and included in the review, with reasons for exclusions at each stage, ideally with a flow diagram.	p.10
Study characteristics	18	For each study, present characteristics for which data were extracted (e.g., study size, PICOS, follow-up period) and provide the citations.	Figure 1, 11
Risk of bias within studies	19	Present data on risk of bias of each study and, if available, any outcome level assessment (see item 12).	Figure 2, p.13
Results of individual studies	20	For all outcomes considered (benefits or harms), present, for each study: (a) simple summary data for each intervention group (b) effect estimates and confidence intervals, ideally with a forest plot.	Table 2, Supplement S6
Synthesis of results	21	Present results of each meta-analysis done, including confidence intervals and measures of consistency.	Table 3, p. 21
Risk of bias across studies	22	Present results of any assessment of risk of bias across studies (see Item 15).	Figure 2, p.14, Supplement S4
Additional analysis	23	Give results of additional analyses, if done (e.g., sensitivity or subgroup analyses, meta-regression [see Item 16]).	Supplement S8, S9

**DISCUSSION**			
Summary of evidence	24	Summarize the main findings including the strength of evidence for each main outcome; consider their relevance to key groups (e.g., healthcare providers, users, and policy makers).	p.22-23
Limitations	25	Discuss limitations at study and outcome level (e.g., risk of bias), and at review-level (e.g., incomplete retrieval of identified research, reporting bias).	p.24
Conclusions	26	Provide a general interpretation of the results in the context of other evidence, and implications for future research.	p.22, p.24
**FUNDING**			
Funding	27	Describe sources of funding for the systematic review and other support (e.g., supply of data); role of funders for the systematic review.	p.26

From: Moher D, Liberati A, Tetzlaff J, Altman DG, The PRISMA Group (2009). Preferred Reporting Items for Systematic Reviews and Meta-Analyses: The PRISMA Statement. PLoS Med 6(7): e1000097. doi:10.1371/journal.pmed1000097

S2: Search strategy

Search conducted in April 2019 with an updated search in October 2020:

(((((("tuberculosis"[mh] OR "mycobacterium tuberculosis"[mh] OR “tuberculosis”[tiab] OR tb[tiab] OR MDRTB[tw] OR "Antitubercular Agents"[mh] OR antitubercul[tw]))) AND ((monitor*[tw] OR infectiousness[tiab] OR treatment outcome*[tiab] OR treatment re-sponse*[tiab] OR “treatment outcome”[mh] OR “microbial viability”[mh] OR viab*[tiab]))) AND ((“biomarkers/blood”[mh] OR biomarker*[tw] OR immunologic test*[tw] OR eth-idium[tw] OR “propidium“[tw] OR “RNA”[tw] OR mRNA[tw] OR “DNA”[tw] OR lympho-cyte*[tw] OR Fluorescein Diacetate[tw] OR (t cell activation marker*[tw] AND assay*) OR gamma assay*[tw]))))*

Table S3: QUADS-2 guiding questions

Domain	Guiding questions
Patient selection	• Was a consecutive or random sample of patients or specimens enrolled?<br>• Was a case-control design avoided?<br>• Did the study avoid inappropriate exclusions?
Reference standard	• Is the reference standard likely to correctly classify the target conditions?<br>• Were the reference standard results interpreted without knowledge of the results of the index test?
Index test	• Were the index test results interpreted without knowledge of the results of the reference standard?<br>• Was the specimen processed as per the manufacturer’s protocol of the particular index test?
Flow and timing	• Was there an appropriate interval between the index tests and reference standard?<br>• Did all patients receive at least one identical reference standard?<br>• Were all patients included in the analysis?

Figure S4: Study-specific QUADAS-2 scores

	Patient selection	Index	Reference	Flow & timing	Overall
Almeida (2009)	X	?	?	+	-
Altunoglu (2014)	+	?	X	+	-
Alzubaidi (2019)	X	?	X	+	X
Andrade (2013)	X	?	?	?	-
Anusiem (2016)	?	?	X	?	-
Atahan (2020)	?	?	?	+	?
Barry (2018)	+	?	?	+	+
Bloom (2012)	?	?	X	X	X
Chedid (2020)	?	?	?	X	-
Chen (2011)	?	?	?	X	-
Choi (2016)	X	?	?	?	-
Chowdhury (2014)	?	?	X	?	-
Chung (2015)	X	?	?	+	-
Chung (2016)	+	?	?	+	+
Darboe (2019)	X	X	?	?	X
Djoba Siawaya (2009)	X	?	?	X	X
Drain (2015)	X	?	?	X	X
Ehtesham (2011)	?	?	X	+	-
Feng (2020)	?	?	?	X	X
Ferrian (2017)	X	?	?	+	-
Feruglio (2013)	?	?	?	+	?
Francisco (2017)	X	?	X	?	X
Garcia-Basteiro (2017)	X	+	?	X	X
Gebremicael (2019a)	?	?	X	?	-
Gebremicael (2019b)	X	X	X	?	X
Hai (2019)	X	?	?	+	-
Honeyborne (2015)	X	?	?	+	-
Honeyborne (2014)	?	?	?	?	?
Honeyborne (2011)	?	?	?	+	?
Hong (2013)	?	?	?	?	?
Ige (2016)	X	?	X	?	X
Jayakumar (2015)	+	+	?	+	+
Jiang (2018)	X	?	?	?	-
Kabeer (2011)	?	X	?	?	-
Kawasaki (2019)	X	+	?	X	X
Khalil (2020)	X	?	?	?	-
Kim (2013)	?	?	?	?	?
Kim (2018)	X	?	?	?	-
Kumar (2017a)	X	?	?	+	-

Judgement:

- X High risk of bias
- \- Intermediate risk of bias
- \+ Low risk of bias
- ? Unclear risk of bias

	Patient selection	Index	Reference	Flow & timing	Overall
Kumar (2016)	X	?	?	+	-
Kumar (2017b)	X	?	?	+	-
Lee (2015)	X	?	?	?	-
Lee (2020)	X	?	X	?	X
Luo (2018)	X	?	X	+	X
Matsushita (2015)	X	?	X	+	X
Mdivani (2009)	?	?	?	?	?
Mendy (2016)	X	?	?	+	-
Mesquita (2016)	X	?	?	?	-
Miranda (2017)	+	?	?	X	-
Montenegro (2014)	?	?	?	+	?
Moraes (2014)	X	?	?	X	X
Mvungi (2019)	X	?	X	?	X
Nie (2020)	?	?	?	+	?
Osawa (2020)	?	?	X	+	-
Penn-Nicholson (2020)	X	?	?	+	-
Rabna (2012)	X	?	X	?	X
Ralph (2013)	X	+	?	X	X
Raras (2010)	?	?	X	+	-
Riou (2012)	?	?	?	+	?
Rockwood (2017)	?	X	X	?	X
Ronacher (2019)	X	?	?	+	-
Sabiiti (2020)	?	?	?	?	?
Sahin (2012)	X	?	?	?	-
Said (2013)	X	X	X	+	X
Siawaya (2008a)	X	+	?	+	-
Siawaya (2008b)	X	?	?	+	-
Sigal (2017)	X	+	?	+	-
Singh (2012)	?	?	?	?	?
Sivakumaran (2020)	?	?	?	?	?
Ugarte-Gil (2013)	?	?	?	+	?
Wang (2016)	X	?	?	+	-
Warsinske (2018)	+	?	?	?	?
Wood (2012)	+	?	X	X	X
Yan (2018)	?	+	?	?	?
Zetola (2016)	X	?	?	+	-
Zhao (2019)	X	?	X	?	X
Zhu (2015)	X	?	X	+	X

Judgement:

- X High risk of bias
- \- Intermediate risk of bias
- \+ Low risk of bias
- ? Unclear risk of bias

Table S5: Complete list of studies and biomarkers included in qualitative analysis

Biomarker	Author (year)	Assay name	Ref	County	Follow ups	Sample used (state)	Reference used
16S rRNA	Hai (2019)	RT-qPCR for 16 S rRNA MBLA on the LightCycler Multiplex RNA Virus Master (Roche)	(1)	Vietnam	0, W1, W2, W4	Sputum (frozen)	Liquid culture (MGIT)<br>Smear microscopy (ZN)<br>GeneXpert (NR)
	Honeyborne (2011)	RT-qPCR for 16S rRNA MBLA on the Rotor-Gene Q 5-plex HRM platform (Qiagen)	(2)	South Africa	0, D3, D7, D14, D56	Sputum (frozen)	Liquid culture (7H9)
	Honeyborne (2014)	16S rRNA MBLA	(3)	Tanzania	0, D2, D4, D5, D7, D10, D14	Sputum (NR)	Solid culture (7H11)<br>Liquid culture (MGIT 960)
	Sabiiti (2020)	RT-qPCR for 16s rRNA MBLA	(4)	Tanzania, Mozambique, Malawi	0, W1, W2, W3, W4, W5, W6, W7, W8, W9, W10, W11, W12	Sputum (NR)	Solid culture (LJ)<br>Liquid culture (MGIT)
	Yan (2018)	Simultaneous amplification and testing for TB (SAT-TB) for 16S rRNA using AmpSure assay (Shanghai Rendu Biotechnology)	(5)	China	0, W2, M2, M5, M6-8	Sputum (NR)	Liquid culture (MGIT 960)<br>Smear microscopy (FM)<br>CT scan
85B mRNA	Atahan (2020)	RT-qPCR for 85B mRNA on the LightCycler 480 II Real-Time PCR System (Roche)	(6)	Turkey	0, D15, D30	Sputum (frozen)	Solid culture (LJ)<br>Liquid culture (MGIT 960)<br>Microscopy (NR)
	Montenegro (2014)	RT-qPCR for 85B mRNA on the ABI PRISM 7500 thermal cycler (Applied Biosystems)	(7)	Brazil	0, D15, D30	Sputum (fresh)	Solid culture (LJ)
	Mdivani (2009)	RT-PCR for 85B mRNA on the ABI PRISM 7700 Sequence Detection System (Applied Biosystems)	(8)	Georgia	0, W2, M1, M2, M4	Sputum (fresh)	Solid culture (LJ)
	Singh (2012)	qRT-PCR for 85B mRNA on the ABI Prism 7000 Sequence Detector System (Perkin Elmer Corp. / Applied Biosystems)	(9)	India	0, D30, D60	NR (NR)	Solid culture (LJ)<br>Liquid culture (BACTEC 460)<br>Smear microscopy (NR)
Adenosine deaminase (ADA)	Altunoglu (2014)	Serum ADA-colorimetric assay (Diazyme General Atomics)	(10)	Turkey	0, M2	Serum (frozen)	Smear microscopy (NR)
	Ige (2016)	ADA ELISA kit (Elabscience)	(11)	Nigeria	0, M6	Plasma (NR)	Smear microscopy (NR)

Albumin	De Moraes (2014)	Albumin Advia colorimetric quantification (Siemens Healthcare Diagnostics)	(12)	Brazil	0, D30, D60	Serum (fresh)	Solid culture (LJ)<br>Clinical outcome<br>Smear microscopy (ZN)
Angiostensinogen	Jiang (2018)	Angiotensinogen ELISA kit (RayBiotech)	(13)	China	0, M2	Serum (NR)	Culture (NR)<br>Smear microscopy (NR)
Angiopoietins (Ang)	Kumar (2017)	Ang-1 and Ang-2 Quantikine ELISA KIT (R&D Systems)	(14)	India	0, M6	Plasma (frozen)	Solid culture (LJ)
Apolipoprotein	Jiang (2018)	Apolipoprotein C-II ELISA kit (RayBiotech)	(13)	China	0, M2	Serum (NR)	Culture (NR)<br>Smear microscopy (NR)
	Jiang (2018)	Apolipoprotein A-IV ELISA kit (CUSABIO Biotech)	(13)	China	0, M2	Serum (NR)	Culture (NR)<br>Smear microscopy (NR)
Beta-2 microglobulin (B2M)	Mendy (2016)	B2M ELISA kit (Immunology Consultants Laboratory)	(15)	Gambia	0, M2, M6	Plasma (frozen)	Liquid culture (MGIT)<br>Smear microscopy (ZN)<br>Chest X-ray
Cancer antigen 125 (CA-125)	Kim (2013)	CA-125 radio-immunoassay kit (Cis Bio International)	(16)	South Korea	0, M6	Serum (NR)	Culture (NR)<br>Smear microscopy (NR)<br>Chest X-ray
	Sahin (2012)	CA-125 microparticle enzyme immunoassay method (Abbott-Axsym System)	(17)	Turkey	0, M2	Serum (NR)	Solid culture (LJ)<br>Smear microscopy (ZN)<br>Chest X-ray
	Said (2013)	VIDAS CA-125 II (Biomerieux)	(18)	Egypt	0, M4	Serum (frozen)	Smear microscopy (ZN)
Complement C7	Jiang (2018)	Complement component C7 ELISA kit (Abcam)	(13)	China	0, M2	Serum (NR)	Culture (NR)<br>Smear microscopy (NR)
Cough frequency	Lee (2020)	CayCaMo cough monitor	(19)	Peru	0, D3, D7, D14, D30, D60	Cough (fresh)	Smear microscopy (NR)<br>Microscopic-observation drug susceptibility (MODS)
C-reactive protein (CRP)	Almeida (2009)	Roche CRPLX kit on the Roche Modular Analyser (Roche)	(20)	Brazil	0, W1, W3, W5, W8	Plasma (frozen)	Solid culture (NR)<br>Clinical outcome<br>Smear microscopy (NR)
	Djoba Siawaya (2008)	CRP ELISA kit (Bender MedSystems)	(21)	South Africa	0, W1, W5, W13, W26	Serum (frozen)	Chest X-ray<br>Liquid culture (BACTEC)
	Ferrian (2017)	CRP on a multiplex (ProcartaPlex Human kits, eBioscience) read on a luminometer (Bioplex 200, BioRad)	(22)	South Africa	0, M2, M4, M6	Plasma (frozen)	Smear microscopy (NR)<br>Liquid culture (MGIT 960)<br>Line probe assay (Hain)

	Francisco (2017	Quantikine ELISA (R&D Systems Inc., Minneapolis, MN, USA)	(23)	China	0, M4, M6, M7	Whole blood (NR)	Clinical outcome
	Jayakumar (2015)	CRP iCHROMA Point-of-care Reader (BodiTech Med Inc)	(24)	Uganda	0, W8, W20	Serum (frozen)	Solid culture (NR)<br>Liquid culture (MGIT 960)
	Khalil (2020)	CRP NycoCARD Reader II (Abbott)	(25)	Egypt	M1, M2, M3	Whole blood (NR)<br>Plasma (NR)<br>Serum (NR)	Solid culture (LJ)<br>Clinical outcome<br>Smear microscopy (ZN)<br>GeneXpert (MTB/RIF)
	Mendy (2016)	CRP ELISA kit (Immunology Consultants Laboratory)	(15)	Gambia	0, M2, M6	Plasma (frozen)	Liquid culture (MGIT)<br>Smear microscopy (ZN)<br>Chest X-ray
	Mesquita (2016)	CRP ELISA kit (Ebioscience)	(26)	Brazil	0, D60	Serum (frozen)	Culture (NR)<br>Chest X-ray
	Miranda (2017)	CRP ELISA kit (Ebioscience)	(27)	Brazil	0, D30, D60	Serum (frozen)	Solid culture (LJ)
	De Moraes (2014)	CRP BNII nephelometer (Dade Behring)	(12)	Brazil	0, D30, D60	Serum (fresh)	Solid culture (LJ)<br>Clinical outcome<br>Smear microscopy (ZN)
	Sigal (2017)	CRP – V-PLEX Human Vascular Injury Panel 2 (Meso Scale Diagnostics)	(28)	North America, Spain, South Africa, Uganda	0, W8, W12	Serum (frozen)	Solid culture (LJ)<br>Liquid culture (MGIT 960)<br>Clinical outcome<br>Chest X-ray
CXCL9	Chung (2015)	CXCL9 ELISA kit (R&D Systems)	(29)	South Korea	0, M1, M2, M3, M6, M12	Serum (frozen)	Culture (NR)<br>Smear microscopy (NR)<br>Clinical outcome<br>Chest X-ray
	Chung (2016)	CXCL9 ELISA kit (R&D Systems)	(30)	South Korea	0, M2	Serum (frozen)	Culture (NR)<br>Smear microscopy (NR)<br>Clinical outcome<br>Chest X-ray
	Lee (2015)	CXCL9 – Bioplex Multiplex Suspension Array System (Bio-Rad Laboratories)	(31)	Taiwan	0, M2	Plasma (NR)<br>Culture supernatant (NR)	Culture (NR)
CXCL11	Chung (2015)	CXCL11 ELISA kit (R&D Systems)	(29)	South Korea	0, M1, M2, M3, M6, M12	Serum (frozen)	Culture (NR)<br>Smear microscopy (NR)<br>Clinical outcome<br>Chest X-ray

	Chung (2016)	CXCL11 ELISA kit (R&D Systems)	(30)	South Korea	0, M2	Serum (frozen)	Culture (NR)<br>Smear microscopy (NR)<br>Clinical outcome<br>Chest X-ray
Endothelin-1 (ET-1)	Wang (2016)	ET-1 ELISA kit (R&D Systems)	(32)	China	0, D1, D2, D4, D6, D10, D14	Sputum (fresh)	Solid culture (7H11)<br>Liquid culture (MGIT 960)
Eotaxin	Choi (2016)	Eotaxin – Millipore Human Cytokine/ Chemokine Magnetic Bead Panel (HCYTOMAG-60 K-02)	(33)	South Korea	0, M2, M6	Serum (fresh)	Solid culture (NR)<br>Liquid culture (NR)<br>Smear microscopy (NR)
	Choi (2016)	CCL11/Eotaxin Quantikine ELISA kit (R&D Systems Inc)	(33)	South Korea	0, M2, M6	Serum (fresh)	Solid culture (NR)<br>Liquid culture (NR)<br>Smear microscopy (NR)
	Djoba Siawaya (2009)	Eotaxin – Lincoplex human cytokine 29-plex assays (Millipore)	(34)	South Africa	0, W1, W5, W13, W26	Plasma (frozen)	Liquid culture (BACTEC 460T)<br>Smear microscopy (NR)
	Riou (2012)	Exotaxin – Multiplex bead array (Bulletin# 10014905 Bio-Rad) on a luminometer (Luminex)	(35)	South Africa	0, W2, W4, W8, W12, W26, W52, W78	Plasma (frozen)	Solid culture (LJ)<br>Liquid culture (MGIT)<br>Smear microscopy (FM)
Epidermal growth factor (EGF)	Djoba Siawaya (2009)	EGF – Lincoplex human cytokine 29-plex assays (Millipore)	(34)	South Africa	0, W1, W5, W13, W26	Plasma (frozen)	Liquid culture (BACTEC 460T)<br>Smear microscopy (NR)
Fractional exhaled nitric oxide (FeNO) & volatile organic compounds (VOCs)	Ralph (2013)	FeNO – Portable NiOX MINO device (Aerocrine, Sweden)	(36)	Indonesia	0, W1, W2, M1, M2, M6	Breath (fresh)	Liquid culture (MGIT)<br>Smear microscopy (NR)<br>Chest X-ray
	Zetola (2016)	VOC – quartz microbalance (QMB) gas sensors	(37)	Botswana	0, D2, D7, D14, D30	Breath (fresh)	Liquid culture (MGIT)
Fibroblast growth factor (FGF) basic	Riou (2012)	FGF basic – Multiplex bead array (Bulletin# 10014905 Bio-Rad) on a luminometer (Luminex)	(35)	South Africa	0, W2, W4, W8, W12, W26, W52, W78	Plasma (frozen)	Solid culture (LJ)<br>Liquid culture (MGIT)<br>Smear microscopy (FM)
Ferritin	Miranda (2017)	Ferritin ELISA kit (Abnova)	(27)	Brazil	0, D30, D60	Serum (frozen)	Solid culture (LJ)
Globulin	De Moraes (2014)	Globulin Advia colorimetric quantification (Siemens Healthcare Diagnostics)	(12)	Brazil	0, D30, D60	Serum (fresh)	Solid culture (LJ)<br>Clinical outcome<br>Smear microscopy (ZN)
Granulocyte colony stimulating factor (G-CSF)	Riou (2012)	G-CSF – Multiplex bead array (Bulletin# 10014905 Bio-Rad) on a luminometer (Luminex)	(35)	South Africa	0, W2, W4, W8, W12, W26, W52, W78	Plasma (frozen)	Solid culture (LJ)<br>Liquid culture (MGIT)<br>Smear microscopy (FM)

Granulocyte macrophage colony stimulating factor (GM-CSF)	Riou (2012)	GM-CSF – Multiplex bead array (Bulletin# 10014905 Bio-Rad) on a luminometer (Luminex))	(35)	South Africa	0, W2, W4, W8, W12, W26, W52, W78	Plasma (frozen)	Solid culture (LJ)<br>Liquid culture (MGIT)<br>Smear microscopy (FM)
Granzyme (A and B)	Djoba Siawaya (2008)	Granzyme B ELISA (Bender MedSystems)	(21)	South Africa	0, W1, W5, W13, W26	Serum (frozen)	Chest X-ray<br>Liquid culture (BACTEC)
	Jayakumar (2015)	Granzyme B ELISA kit (Bender MedSystems)	(24)	Uganda	0, W8, W20	Serum (frozen)	Solid culture (NR)<br>Liquid culture (MGIT 960)
	Lee (2015)	Granzyme A – Bioplex Multiplex Suspension Array System (Bio-Rad Laboratories)	(31)	Taiwan	0, M2	Plasma (NR)<br>Culture supernatant (NR)	Culture (NR)
Heme oxygenase-1 (HO-1)	Andrade (2013)	HO-1 ELISA kit (Assay Designs)	(38)	India	W0, W26	Plasma (NR)	Culture (NR)
	Rockwood (2017)	HO-1 ELISA kit (ADI-EKS-800, Enzo Life Sciences)	(39)	South Africa	0, W8, W20	Plasma (frozen)	Culture (NR)<br>Chest X-ray
Hemoglobin	De Moraes (2014)	Hemoglobin ABX Pentra DF 120 (Horiba Diagnostics)	(12)	Brazil	0, D30, D60	Serum (fresh)	Solid culture (LJ)<br>Clinical outcome<br>Smear microscopy (ZN)
High-sensitivity CRP (hs-CRP)	Altunoglu (2014)	hs-CRP BN II nephelometer (Dade Behring)	(10)	Turkey	0, M2	Serum (frozen)	Smear microscopy (NR)
	Chen (2011)	hs-CRP nephelometry (Dade Behring BM2)	(40)	Taiwan	0, M2, M6	Serum (NR)	Solid culture (LJ)<br>Liquid culture (MGIT)<br>Smear microscopy (ZN)<br>Chest X-ray
	Ehtesham (2011)	hs-CRP ELISA kit (Diagnostic Biochem Canada Inc.)	(41)	India	0, M2, M4, M6	Serum (frozen)	Clinical outcome<br>Smear microscopy (NR)
IFN-γ	Chowdhury (2014)	IFN-γ high sensitivity human ELISA kit (ImmunoTools)	(42)	India	0, M2, M4, M6	Serum (NR)	Smear microscopy (ZN)<br>Chest X-ray
	Chung (2015)	IFN-y ELISA kit (R&D Systems)	(29)	South Korea	0, M1, M2, M3, M6, M12	Serum (frozen)	Culture (NR)<br>Smear microscopy (NR)<br>Clinical outcome<br>Chest X-ray
	Djoba Siawaya (2008)	IFN-γ – Bio-Plex bead array system (Bio-Rad Laboratories)	(43)	South Africa	0, D3, W1, W2, W4, W8, W13, W26	Peripheral blood (frozen)	Smear microscopy (ZN)<br>Liquid culture (BACTEC 460TB)
	Nie (2020)	IFN-γ ELISA kit (BioLegend)	(44)	China	0, M1-2, M6	Serum (frozen)	Culture (NR)<br>Smear microscopy (NR)<br>CT scan

	Matsushita (2015)	IFN-γ – 27-plex assay on the Bio-Plex Suspension Array System (Bio-Rad)	(45)	Vietnam	0, M2, M7	Plasma (frozen)	Smear microscopy (NR)<br>Chest X-ray
	Mvungi (2019)	IFN-γ – multiplex assay (Human Premixed Multi-Analyte Kit; cat. # LXSAHM) on the Luminex 200 system	(46)	Tanzania	0, M2	Plasma (frozen) prepared using QuantiFERON-TB Gold Plus	Clinical outcome
	Riou (2012)	IFN-γ – Multiplex bead array (Bulletin# 10014905 Bio-Rad) on a luminometer (Luminex)	(35)	South Africa	0, W2, W4, W8, W12, W26, W52, W78	Plasma (frozen)	Solid culture (LJ)<br>Liquid culture (MGIT)<br>Smear microscopy (FM)
	Sigal (2017)	IFN-γ – V-PLEX Human Pro-inflammatory Panel 1 (Meso Scale Diagnostics)	(28)	North America, Spain, South Africa, Uganda	0, W8, W12	Serum (frozen)	Solid culture (LJ)<br>Liquid culture (MGIT 960)<br>Clinical outcome<br>Chest X-ray
Iron	Gebremicael (2019)	Iron on c501 module of cobas6000 analyser (Roche Diagnostics)	(47)	Ethiopia	0, M6	Serum (frozen)	Clinical outcome<br>Smear microscopy (ZN)
IS6110 insertion element	Mdivani (2009)	RT-DNA for IS6110 insertion using ABI PRISM 7700 Sequence Detection System (Applied Biosystems)	(8)	Georgia	0, W2, M1, M2, M4	Sputum (fresh)	Solid culture (LJ)
IL-1β	Anusiem (2016)	Interleukin-1β ELISA kit (eBioscience)	(48)	Nigeria	W0, W8, W26	Serum (frozen)	Smear microscopy (NR)
	Chowdhury (2014)	Human IL-1β ELISA kit (Raybiotech)	(42)	India	0, M2, M4, M6	Serum (NR)	Smear microscopy (ZN)<br>Chest X-ray
	Luo (2018)	IL-1β ELISA kits (Siemens Healthcare Diagnostics)	(49)	China	0, M2	Serum (fresh)	Smear microscopy (FM)
	Riou (2012)	IL-1β – Multiplex bead array (Bulletin# 10014905 Bio-Rad) on a luminometer (Luminex)	(35)	South Africa	0, W2, W4, W8, W12, W26, W52, W78	Plasma (frozen)	Solid culture (LJ)<br>Liquid culture (MGIT)<br>Smear microscopy (FM)
IL-1RA	Matsushita (2015)	IL-1RA – 27-plex assay on the Bio-Plex Suspension Array System (Bio-Rad)	(45)	Vietnam	0, M2, M7	Plasma (frozen)	Smear microscopy (NR)<br>Chest X-ray
	Riou (2012)	IL-1RA – Multiplex bead array (Bulletin# 10014905 Bio-Rad) on a luminometer (Luminex)	(35)	South Africa	0, W2, W4, W8, W12, W26, W52, W78	Plasma (frozen)	Solid culture (LJ)<br>Liquid culture (MGIT)<br>Smear microscopy (FM)

IL-2	Alzubaidi (2019)	IL-2 ELISA kit (R&D Systems)	(50)	Iraq	W0, D2, D4, W1, W2, W3, W4, W9	Sputum (fresh)	Smear microscopy (NR)
	Kumar (2017)	IL-2 Quantikine ELISA kit (R&D Systems)	47	India	0, M6	Plasma (fresh)	Culture (NR)
	Matsushita (2015)	IL-2 – 27-plex assay on the Bio-Plex Suspension Array System (Bio-Rad)	(45)	Vietnam	0, M2, M7	Plasma (frozen)	Smear microscopy (NR)<br>Chest X-ray
	Riou (2012)	IL-2 – Multiplex bead array (Bulletin# 10014905 Bio-Rad) on a luminometer (Luminex)	(35)	South Africa	0, W2, W4, W8, W12, W26, W52, W78	Plasma (frozen)	Solid culture (LJ)<br>Liquid culture (MGIT)<br>Smear microscopy (FM)
IL-4	Chowdhury (2014)	IL-4 high sensitivity human ELISA kit (ImmunoTools)	(42)	India	0, M2, M4, M6	Serum (NR)	Smear microscopy (ZN)<br>Chest X-ray
	Djoba Siawaya (2008)	IL4 – Bio-Plex bead array system (Bio-Rad Laboratories)	(43)	South Africa	0, D3, W1, W2, W4, W8, W13, W26	Peripheral blood (frozen)	Smear microscopy (ZN)<br>Liquid culture (BACTEC 460TB)
	Mvungi (2019)	IL-4 multiplex assay (Human Premixed Multi-Analyte Kit; cat. # LXSAHM) on the Luminex 200 system	(46)	Tanzania	0, M2	Plasma (frozen)	Clinical outcome
	Nie (2020)	IL-4 ELISA kit (BioLegend, USA)	(44)	China	0, M1-2, M6	Serum (frozen)	Culture (NR)<br>Smear microscopy (NR)<br>Chest computed tomography
	Riou (2012)	IL-4 – Multiplex bead array (Bulletin# 10014905 Bio-Rad) on a luminometer (Luminex)	(35)	South Africa	0, W2, W4, W8, W12, W26, W52, W78	Plasma (frozen)	Solid culture (LJ)<br>Liquid culture (MGIT)<br>Smear microscopy (FM)
IL-5	Riou (2012)	IL-5 – Multiplex bead array (Bulletin# 10014905 Bio-Rad) on a luminometer (Luminex)	(35)	South Africa	0, W2, W4, W8, W12, W26, W52, W78	Plasma (frozen)	Solid culture (LJ)<br>Liquid culture (MGIT)<br>Smear microscopy (FM)
IL-6	Chowdhury (2014)	IL-6 ELISA kit (Raybiotech)	(42)	India	0, M2, M4, M6	Serum (NR)	Smear microscopy (ZN)<br>Chest X-ray
	Djoba Siawaya (2009)	IL-6 – Lincoplex human cytokine 29-plex assays (Millipore)	(34)	South Africa	0, W1, W5, W13, W26	Plasma (frozen)	Liquid culture (BACTEC 460T)<br>Smear microscopy (NR)
	Feng (2020)	IL-6 Quantikine ELISA kit (R&D Systems)	(51)	Taiwan	0, W8	Peripheral blood mononuclear cells (frozen)	Culture (NR)<br>Smear microscopy (NR)

	Luo (2018)	IL-6 ELISA kits (Siemens Healthcare Diagnostics Products Ltd., Llanberis, Gwynedd, UK)	(49)	China	0, M2	Serum (fresh)	Smear microscopy (FM)
	Mvungi (2019)	IL-6 multiplex assay (Human Premixed Multi-Analyte Kit; cat. # LXSAHM) on the Luminex 200 system	(46)	Tanzania	0, M2	Plasma (frozen) prepared using QuantiFERON-TB Gold Plus	Clinical outcome
	Riou (2012)	IL-6 – Multiplex bead array (Bulletin# 10014905 Bio-Rad) on a luminometer (Luminex)	(35)	South Africa	0, W2, W4, W8, W12, W26, W52, W78	Plasma (frozen)	Solid culture (LJ)<br>Liquid culture (MGIT)<br>Smear microscopy (FM)
	Sigal (2017)	IL-6 – V-PLEX Human Pro-inflammatory Panel 1 (Meso Scale Diagnostics)	(28)	North America, Spain, South Africa, Uganda	0, W8, W12	Serum (frozen)	Solid culture (LJ)<br>Liquid culture (MGIT 960)<br>Clinical outcome<br>Chest X-ray
IL-7	Kumar (2017)	IL-7 Quantikine ELISA kit (R&D Systems)	(52)	India	0, M6	Plasma (fresh)	Culture (NR)
	Riou (2012)	IL-7 – Multiplex bead array (Bulletin# 10014905 Bio-Rad) on a luminometer (Luminex)	(35)	South Africa	0, W2, W4, W8, W12, W26, W52, W78	Plasma (frozen)	Solid culture (LJ)<br>Liquid culture (MGIT)<br>Smear microscopy (FM)
IL-9	Riou (2012)	IL-9 – Multiplex bead array (Bulletin# 10014905 Bio-Rad) on a luminometer (Luminex)	(35)	South Africa	0, W2, W4, W8, W12, W26, W52, W78	Plasma (frozen)	Solid culture (LJ)<br>Liquid culture (MGIT)<br>Smear microscopy (FM)
IL-10, IL-10Ra	Chowdhury (2014)	IL-10 high sensitivity human ELISA set (ImmunoTools)	(42)	India	0, M2, M4, M6	Serum (NR)	Smear microscopy (ZN)<br>Chest X-ray
	Djoba Siawaya (2009)	IL-10 – Lincoplex human cytokine 29-plex assays (Millipore)	(34)	South Africa	0, W1, W5, W13, W26	Plasma (frozen)	Liquid culture (BACTEC 460T)<br>Smear microscopy (NR)
	Mvungi (2019)	IL-10 – multiplex assay (Human Premixed Multi-Analyte Kit; cat. # LXSAHM) on the Luminex 200 system	(46)	Tanzania	0, M2	Plasma (frozen) prepared using QuantiFERON-TB Gold Plus	Clinical outcome
	Riou (2012)	IL-10 – Multiplex bead array (Bulletin# 10014905 Bio-Rad) on a luminometer (Luminex)	(35)	South Africa	0, W2, W4, W8, W12, W26, W52, W78	Plasma (frozen)	Solid culture (LJ)<br>Liquid culture (MGIT)<br>Smear microscopy (FM)
	Zhao (2019)	IL-10Ra ELISA kit (accession #: Q13651; RayBiotech)	(53)	China	0, W1, W2, W3, W4, W5, W6, W7, W8	Peripheral blood mononuclear cells (fresh)	Smear microscopy (NR)

IL-12, IL-12p40, IL-12p700	Djoba Siawaya (2009)	IL-12p40 – Lincoplex human cytokine 29-plex assays (Millipore)	(34)	South Africa	0, W1, W5, W13, W26	Plasma (frozen)	Liquid culture (BACTEC 460T)<br>Smear microscopy (NR)
	Chowdhury (2014)	IL-12p40 ELISA Kit (RayBiotech)	(42)	India	0, M2, M4, M6	Serum (NR)	Smear microscopy (ZN)<br>Chest X-ray
	Riou (2012)	IL-12p70 – Multiplex bead array (Bulletin# 10014905 Bio-Rad) on a luminometer (Luminex))	(35)	South Africa	0, W2, W4, W8, W12, W26, W52, W78	Plasma (frozen)	Solid culture (LJ)<br>Liquid culture (MGIT)<br>Smear microscopy (FM)
	Zhu (2015)	IL-12 – ELISA kit (eBioscience)	(54)	China	0, W2-8	Plasma (frozen)	Smear microscopy (NR)
IL-13	Djoba Siawaya (2009)	IL-13 – Lincoplex human cytokine 29-plex assays (Millipore)	(34)	South Africa	0, W1, W5, W13, W26	Plasma (frozen)	Liquid culture (BACTEC 460T)
	Riou (2012)	IL-13 – Multiplex bead array (Bulletin# 10014905 Bio-Rad) on a luminometer (Luminex)	(35)	South Africa	0, W2, W4, W8, W12, W26, W52, W78	Plasma (frozen)	Solid culture (LJ)<br>Liquid culture (MGIT)<br>Smear microscopy (FM)
IL-15	Djoba Siawaya (2009)	IL-15 – Lincoplex human cytokine 29-plex assays (Millipore)	(34)	South Africa	0, W1, W5, W13, W26	Plasma (frozen)	Liquid culture (BACTEC 460T)<br>Smear microscopy (NR)
	Kumar (2017)	IL-15 Quantikine ELISA kit (R&D Systems)	(52)	India	0, M6	Plasma (fresh)	Culture (NR)
	Riou (2012)	IL-15 – Multiplex bead array (Bulletin# 10014905 Bio-Rad) on a luminometer (Luminex)	(35)	South Africa	0, W2, W4, W8, W12, W26, W52, W78	Plasma (frozen)	Solid culture (LJ)<br>Liquid culture (MGIT)<br>Smear microscopy (FM)
IL-17	Chen (2011)	IL-17 ELISA (R&D Systems)	(40)	Taiwan	0, M2, M6	Serum (NR)	Solid culture (LJ)<br>Liquid culture (MGIT)<br>Smear microscopy (ZN)<br>Chest X-ray
	Riou (2012)	IL-17 – Multiplex bead array (Bulletin# 10014905 Bio-Rad) on a luminometer (Luminex)	(35)	South Africa	0, W2, W4, W8, W12, W26, W52, W78	Plasma (frozen)	Solid culture (LJ)<br>Liquid culture (MGIT)<br>Smear microscopy (FM)
IL-17A	Feng (2020)	IL-17A – 7-Plex Human ProcartaPlex Panel (Affymetrix eBioscience)	(51)	Taiwan	0, W8	Peripheral blood (frozen)	Culture (NR)<br>Smear microscopy (NR)
IL-18	Lee (2015)	IL-18 – Bioplex Multiplex Suspension Array System (Bio-Rad Laboratories)	(31)	Taiwan	0, M2	Plasma (NR)<br>Culture supernatant (NR)	Culture (NR)

IL-21	Feng (2020)	IL-21 – 7-Plex Human ProcartaPlex Panel (Affymetrix eBioscience)	(51)	Taiwan	0, W8	Peripheral blood (frozen)	Culture (NR)<br>Smear microscopy (NR)
	Kumar (2017)	IL-21 Quantikine ELISA kit (R&D Systems)	(52)	India	0, M6	Plasma (fresh)	Culture (NR)
IL-22	Feng (2020)	IL-22 – 7-Plex Human ProcartaPlex Panel (Affymetrix eBioscience)	(51)	Taiwan	0, W8	Peripheral blood (frozen)	Culture (NR)<br>Smear microscopy (NR)
	Lee (2015)	IL-22 – Bioplex Multiplex Suspension Array System (Bio-Rad Laboratories)	(31)	Taiwan	0, M2	Plasma (NR)<br>Culture supernatant (NR)	Culture (NR)
IL-23	Feng (2020)	IL-23 – 7-Plex Human ProcartaPlex Panel (Affymetrix eBioscience)	(51)	Taiwan	0, W8	Peripheral blood (frozen)	Culture (NR)<br>Smear microscopy (NR)
IP-10 (CXCL10)	Chen (2011)	IP-10 ELISA (R&D Systems)	(40)	Taiwan	0, M2, M6	Serum (NR)	Solid culture (LJ)<br>Liquid culture (MGIT)<br>Smear microscopy (ZN)<br>Chest X-ray
	Chung (2016)	IP-10 ELISA (R&D systems, Minneapolis, MN)	(30)	South Korea	0, M2	Serum (frozen)	Culture (NR)<br>Smear microscopy (NR)<br>Clinical outcome<br>Chest X-ray
	Djoba Siawaya (2009)	IP-10 – Lincoplex human cytokine 29-plex assays (Millipore)	(34)	South Africa	0, W1, W5, W13, W26	Plasma (frozen)	Liquid culture (BACTEC 460T)<br>Smear microscopy (NR)
	Ferrian (2017)	IP-10 on a multiplex (ProcartaPlex Human kits, eBioscience) read on a luminometer (Bioplex 200, BioRad)	(22)	South Africa	0, M2, M4, M6	Plasma (frozen)	Smear microscopy (NR)<br>Liquid culture (MGIT 960)
	Francisco (2017)	ELISA kit (Raybiotech, Inc.)	(23)	China	0, M4, M6, M7	Whole blood (NR)	Clinical outcome
	Garcia-Basteiro (2017)	IP-10 ELISA kit (Becton Dickinson and Company)	(55)	Mozambique	0, D7, D60	Serum (frozen)	Smear microscopy (ZN)<br>GeneXpert (MTB/RIF)<br>Liquid culture (MGIT 960)
	Hong (2013)	IP-10 ELISA kit (R&D Systems)	(56)	South Korea	0/within W2, after M6-M9	Serum (NR)	Culture (NR)<br>Chest X-ray<br>CT scan

	Jayakumar (2015)	IP-10 ELISA kit (R&D Systems)	(24)	Uganda	0, W8, W20	Serum (frozen)	Solid culture (NR)<br>Liquid culture (MGIT 960)
	Kabeer (2011)	IP-10 ELISA kit (R&D Sysytems) in response to QFT-IT and RD1	(57)	India	0, M6	Plasma (fresh)	Solid culture (LJ)<br>Liquid culture (BacT)
	Kim (2018)	IP-10 ELISA kit (R&D Systems, Minneapolis, MN, USA)	(58)	South Korea	0, M6, M12	Urine (NR), Serum (NR)	Culture (NR)<br>Chest X-ray
	Matsushita (2015)	IP-10 – 27-plex assay on the Bio-Plex Suspension Array System (Bio-Rad)	(45)	Vietnam	0, M2, M7	Plasma (frozen)	Smear microscopy (NR)<br>Chest X-ray
	Riou (2012)	IP-10 – Multiplex bead array (Bulletin# 10014905 Bio-Rad) on a luminometer (Luminex)	(35)	South Africa	0, W2, W4, W8, W12, W26, W52, W78	Plasma (frozen)	Solid culture (LJ)<br>Liquid culture (MGIT)<br>Smear microscopy (FM)
	Zhu (2015)	IP-10 ELISA kit (eBioscience)	(54)	China	0, W2-8	Plasma (frozen)	Smear microscopy (NR)
KL-6	Djoba Siawaya (2009)	KL-6 ELISA kit (Sanko Junyaku)	(34)	South Africa	0, W1, W5, W13, W26	Plasma (frozen)	Liquid culture (BACTEC 460T)
Leukocyte immunoglobulin like receptor B4 (LILRB4)	Zhao (2019)	SLAMF8 ELISA kit (accession #: Q8NHJ6; RayBiotech)	(53)	China	0, W1, W2, W3, W4, W5, W6, W7, W8	Peripheral blood mononuclear cells (fresh)	Smear microscopy (NR)
Lipoarabinomannan (LAM)	Drain (2015)	Determine TB LAM test (Alere Inc.)	(59)	South Africa	0, M2, EOT	Urine (frozen)	Liquid culture (MGIT)<br>Solid culture (NR)<br>Clinical outcome
	Kawasaki (2019)	LAM ELISA kit (Otsuka Pharmaceuticals)	(60)	Philippines	0, D7, D14, D28, D56	Sputum (NR)	Liquid culture (MGIT)<br>Smear microscopy (NR)<br>TB-LAMP
	Wood (2012)	LAM ELISA kit (Alere Health Services)	(61)	South Africa	0, W1, W2, W8, W16, W24	Urine (frozen)	GeneXpert (MTB/RIF)
	Feruglio (2013)	Limulus amebocyte lysate colorimetric assay (Lonza) to detect TB-LAM from the LAM ELISA kit (Wuhan Eiaab Science)	(62)	Norway	0, W2, W8, W24	Plasma (frozen)	Culture (NR)<br>Smear microscopy (NR)
Lipocalcin-2	Choi (2016)	Lipocalcin-2 – Millipore Human Cytokine/ Chemokine Magnetic Bead Panel (HCYTOMAG-60 K-02)	(33)	South Korea	0, M2, M6	Serum (fresh)	Solid culture (NR)<br>Liquid culture (NR)<br>Smear microscopy (NR)
	Choi (2016)	MIP-1α – Millipore Human Cytokine/ Chemokine Magnetic	(33)	South Korea	0, M2, M6	Serum (fresh)	Solid culture (NR)<br>Liquid culture (NR)

		Bead Panel (HCYTOMAG-60 K-02)					Smear microscopy (NR)
Macrophage inflammatory proteins (MIP)	Djoba Siawaya (2009)	MIP-1α & MIP-1β – Lincoplex human cytokine 29-plex assays (Millipore)	(34)	South Africa	0, W1, W5, W13, W26	Plasma (frozen)	Liquid culture (BACTEC 460T)<br>Smear microscopy (NR)
	Riou (2012)	MIP-1β – Multiplex bead array (Bulletin# 10014905 Bio-Rad) on a luminometer (Luminex)	(35)	South Africa	0, W2, W4, W8, W12, W26, W52, W78	Plasma (frozen)	Solid culture (LJ)<br>Liquid culture (MGIT)<br>Smear microscopy (FM)
Matrix metalloproteinase (MMP)	Lee (2015)	MMP-8 – Bioplex Multiplex Suspension Array System (Bio-Rad Laboratories)	(31)	Taiwan	0, M2	Plasma (NR)<br>Culture supernatant (NR)	Culture (NR)
	Sigal (2017)	MMP-8 – TB Panel 4 custom designed (Meso Scale Diagnostics)	(28)	North America, Spain, South Africa, Uganda	0, W8, W12	Serum (frozen)	Solid culture (LJ)<br>Liquid culture (MGIT 960)<br>Clinical outcome<br>Chest X-ray
	Ugarte-Gil (2013)	MMP-1, -2, -3, -7, -8, -9 using Luminex multiplex array (R&D Systems)	(63)	Peru	0, W2, W8, W24	Sputum (frozen)	Liquid culture (NR)<br>Clinical outcome<br>Smear microscopy (FM)
Monocyte chemotactic protein1 (MCP-1)	Djoba Siawaya (2009)	MCP-1 – Lincoplex human cytokine 29-plex assays (Millipore)	(34)	South Africa	0, W1, W5, W13, W26	Plasma (frozen)	Liquid culture (BACTEC 460T)<br>Smear microscopy (NR)
	Ferrian (2017)	MCP-1 on a multiplex (ProcartaPlex Human kits, eBioscience) read on a luminometer (Bioplex 200, BioRad)	(22)	South Africa	0, M2, M4, M6	Plasma (frozen)	Smear microscopy (NR)<br>Liquid culture (MGIT 960)<br>Line probe assay (Hain)
	Riou (2012)	MCP-1 – Multiplex bead array (Bulletin# 10014905 Bio-Rad) on a luminometer (Luminex)	(35)	South Africa	0, W2, W4, W8, W12, W26, W52, W78	Plasma (frozen)	Solid culture (LJ)<br>Liquid culture (MGIT)<br>Smear microscopy (FM)
Neopterin	Altunoglu (2014)	Neopterin ELISA KIT (Immuno-Biological Laboratories)	(10)	Turkey	0, M2	Serum (frozen)	Smear microscopy (NR)
	Jayakumar (2015)	Neopterin ELISA kit (Immuno-Biological Laboratories)	(24)	Uganda	W0, W8, W20	Serum (frozen)	Solid culture (NR)<br>Liquid culture (MGIT 960)
	Mendy (2016)	Neopterin ELISA kit (Immuno-Biological Laboratories)	(15)	Gambia	0, M2, M6	Plasma (frozen)	Liquid culture (MGIT)<br>Smear microscopy (ZN)<br>Chest X-ray
Osteopontin (OPN)	Zhu (2015)	OPN ELISA kit (R&D Systems)	(54)	China	0, W2-8	Plasma (frozen)	Smear microscopy (NR)

Pentraxin-3 (PTX-3)	Sigal (2017)	PTX-3 – TB Panel 2 custom designed (Meso Scale Diagnostics)	(28)	North America, Spain, South Africa, Uganda	0, W8, W12	Serum (frozen)	Solid culture (LJ)<br>Liquid culture (MGIT 960)<br>Clinical outcome<br>Chest X-ray
Procalcitonin	Osawa (2020)	µTASWako i30 immunoanalyzer (Fujifilm Wako Pure Chemical)	(64)	South Africa	0, D7, D14, D28	Serum (NR)	Smear microscopy (NR)<br>Chest X-ray
Platelet derived growth factor (PDGF) BB	Riou (2012)	PDGF-BB – Multiplex bead array (Bulletin# 10014905 Bio-Rad) on a luminometer (Luminex)	(35)	South Africa	0, W2, W4, W8, W12, W26, W52, W78	Plasma (frozen)	Solid culture (LJ)<br>Liquid culture (MGIT)<br>Smear microscopy (FM)
RANTES	Lee (2015)	RANTES – Bioplex Multiplex Suspension Array System (Bio-Rad Laboratories)	(31)	Taiwan	0, M2	Plasma (NR)<br>Culture supernatant (NR)	Culture (NR)
Resistin	Ehtesham (2011)	Human resistin ELISA kit (AdipoGen)	(41)	India	0, M2, M4, M6	Serum (frozen)	Clinical outcome
sCD14	Feruglio (2013)	sCD14 ELISA kit (R&D Systems)	(62)	Norway	0, W2, W8, W24	Plasma (frozen)	Culture (NR)<br>Smear microscopy (NR)
sCD40L	Djoba Siawaya (2009)	sCD40L – Lincoplex human cytokine 29-plex assays (Millipore)	(34)	South Africa	0, W1, W5, W13, W26	Plasma (frozen)	Liquid culture (BACTEC 460T)<br>Smear microscopy (NR)
sICAM-1	Djoba Siawaya (2008)	sICAM-1 ELISA kit (R&D Systems)	(21)	South Africa	0, W1, W5, W13, W26	Serum (frozen)	Chest X-ray<br>Liquid culture (BACTEC)
	Jayakumar (2015)	sICAM-1 ELISA kit (R&D Systems)	(24)	Uganda	0, W8, W20	Serum (frozen)	Solid culture (NR)<br>Liquid culture (MGIT 960)
Serum amyloid A (SAA) and serum amyloid protein A (SAA1)	Ferrian (2017)	SAA on a multiplex (ProcartaPlex Human kits, eBioscience) read on a luminometer (Bioplex 200, BioRad)	(22)	South Africa	0, M2, M4, M6	Plasma (frozen)	Smear microscopy (NR)<br>Liquid culture (MGIT 960)<br>Line probe assay (Hain)
	Sigal (2017)	SAA1 – V-PLEX Human Vascular Injury Panel 2 (Meso Scale Diagnostics)	(28)	North America, Spain, South Africa, Uganda	0, W8, W12	Serum (frozen)	Solid culture (LJ)<br>Liquid culture (MGIT 960)<br>Clinical outcome<br>Chest X-ray
SLAMF8	Zhao (2019)	SLAMF8 ELISA kit (accession #: Q9P0V8; RayBiotech)	(53)	China	0, W1, W2, W3, W4, W5, W6, W7, W8	Peripheral blood mononuclear cells (fresh)	Smear microscopy (NR)
Small RNA (smRNA) and microRNA (miRNA)	Barry (2018)	5 plasma miRNA<br>10 plasma miRNA	(65)	China	0, M1, M2, M6	Plasma (frozen)	Culture (NR)<br>Smear microscopy (NR)<br>Chest X-ray<br>Clinical outcome

	Honeyborne (2015)	miR-29A, SNORD61, miR-17-3p, miR-133a using Rotorgene-100 human serum/plasma MIHS-106Z arrays (SABiosciences)	(66)	South Africa	0, W24	Plasma (frozen)	Culture (NR)
Soluble death receptor (sDR)	Djoba Siawaya (2008)	sDR5 – BioPlex bead array system (Bio-Rad Laboratories) on Luminex	(21)	South Africa	0, W1, W5, W13, W26	Serum (frozen)	Chest X-ray<br>Liquid culture (BACTEC)
Soluble lymphocyte activation gene-3 (sLAG-3)	Djoba Siawaya (2008)	sLAG-3 ELISA kit (Apotech)	(21)	South Africa	0, W1, W5, W13, W26	Serum (frozen)	Chest X-ray<br>Liquid culture (BACTEC)
Soluble receptor analytes	Choi (2016)	sIL-2Ra – Millipore Human Cytokine/ Chemokine Magnetic Bead Panel (HCYTOMAG-60 K-02)	(33)	South Korea	0, M2, M6	Serum (fresh)	Solid culture (NR)<br>Liquid culture (NR)<br>Smear microscopy (NR)
	Ferrian (2017)	sIL2Ra on a multiplex (ProcartaPlex Human kits, eBioscience) read on a luminometer (Bioplex 200, BioRad)	(22)	South Africa	0, M2, M4, M6	Plasma (frozen)	Smear microscopy (NR)<br>Liquid culture (MGIT 960)<br>Line probe assay (Hain)
	Jayakumar (2015)	sIL-2Ra, sTNF-R1, and sTNF-R2 on the Luminex 100 multiplex platform (Millipore Corporation)	(24)	Uganda	0, W8, W20	Serum (frozen)	Solid culture (NR)<br>Liquid culture (MGIT 960)
	Luo (2018)	sIL-2R ELISA kit (Siemens Healthcare Diagnostics)	(49)	China	0, M2	Serum (fresh)	Smear microscopy (FM)
	Nie (2020)	sIL-2R ELISA kit (BioLegend)	(44)	China	0, M1-2, M6	Serum (frozen)	Culture (NR)<br>Smear microscopy (NR)<br>CT scan
Soluble tumor necrosis factor receptor (sTNFR)	Djoba Siawaya (2008)	sTNF I, sTNFR II – BioPlex bead array system (Bio-Rad Laboratories) on Luminex	(21)	South Africa	0, W1, W5, W13, W26	Serum (frozen)	Chest X-ray<br>Liquid culture (BACTEC)
Soluble urokinase-type plasminogen activator receptor (suPAR)	Jayakumar (2015)	suPAR ELISA kit (ViroGates)	(24)	Uganda	0, W8, W20	Serum (frozen)	Solid culture (NR)<br>Liquid culture (MGIT 960)
	Rabna (2012)	suPAR ELISA kit (ViroGates)	(67)	Guinea-Bissau	0, W2, M1, M2, M5, M8	Plasma (frozen)	Clinical outcome<br>Smear microscopy (ZN)
	Raras (2010)	suPAR ELISA kit (ViroGates)	(68)	Indonesia	0, M2, M4, M6	Serum (frozen)	Clinical outcome<br>Smear microscopy (NR)<br>Chest X-ray

	Ronacher (2019)	suPAR ELISA kit (Virogates)	(69)	South Africa	0, W1, W2, W4, W6 W26	Serum (frozen)	Liquid culture (Bactec 460)
	Djoba Siawaya (2008)	suPAR ELISA kit (ViroGates)	(21)	South Africa	0, W1, W5, W13, W26	Serum (frozen)	Chest X-ray<br>Liquid culture (BACTEC)
TGF-β1	Chowdhury (2014)	TGF-β1 ELISA Kit (RayBiotech)	(42)	India	0, M2, M4, M6	Serum (NR)	Smear microscopy (ZN)<br>Chest X-ray
Tissue inhibitor metalloproteinase (TIMP)	Ugarte-Gil (2013)	TIMP-1 & TIMP-2 using ELISA kit (R&D Systems)	(63)	Peru	0, W2, W8, W24	Sputum (frozen)	Liquid culture (NR)<br>Clinical outcome<br>Smear microscopy (FM)
TNF-α	Chowdhury (2014)	TNF-α high sensitivity human ELISA set (ImmunoTools)	(42)	India	0, M2, M4, M6	Serum (NR)	Smear microscopy (ZN)<br>Chest X-ray
	Djoba Siawaya (2009)	TNF-α – Lincoplex human cytokine 29-plex assays (Millipore)	(34)	South Africa	0, W1, W5, W13, W26	Plasma (frozen)	Liquid culture (BACTEC 460T)<br>Smear microscopy (NR)
	Luo (2018)	TNF-α ELISA kit (Siemens Healthcare Diagnostics)	(49)	China	0, M2	Serum (fresh)	Smear microscopy (FM)
	Mvungi (2019)	TNF-α – multiplex assay (Human Premixed Multi-Analyte Kit; cat. # LXSAHM) on the Luminex 200 system	(46)	Tanzania	0, M2	Plasma (frozen) prepared using QuantiFERON-TB Gold Plus	Clinical outcome
	Nie (2020)	TNF-α ELISA kit (BioLegend)	(44)	China	0, M1-2, M6	Serum (frozen)	Culture (NR)<br>Smear microscopy (NR)<br>Chest computed tomography
	Riou (2012)	TNF-α – Multiplex bead array (Bulletin# 10014905 Bio-Rad) on a luminometer (Luminex))	(35)	South Africa	0, W2, W4, W8, W12, W26, W52, W78	Plasma (frozen)	Solid culture (LJ)<br>Liquid culture (MGIT)<br>Smear microscopy (FM)
	Zhu (2015)	TNF-α ELISA kit (eBioscience)	(54)	China	0, W2-8	Plasma (frozen)	Smear microscopy (NR)
Transcriptomic/ gene signatures	Bloom (2012)	664-transcript signature<br>320-transcript signature	(70)	South Africa	0, W2, M2, M6, M12	Whole blood (frozen)	Clinical outcome<br>Chest X-ray
	Darboe (2019)	RISK11 signature	(71)	South Africa	0, M2, M6, M8,M14	Whole blood (NR)	Culture (NR)
	Francisco (2017)	3-gene signature (*GBP5*, *DUSP3* and *KLF2*)	(23)	China	0, M4, M6, M7	Whole blood (NR)	Clinical outcome
	Gebremicael (2019)	105 genes expression profiling by dual-color Reverse-Transcription Multiplex Ligation-dependent	(72)	Ethiopia	0, M6, M18	Whole blood (NR)	Smear microscopy (ZN)

		Probe Amplification (dcRT-MLPA) platform					
	Penn-Nicholson (2020)	RISK6 signature	(73)	South Africa	0, M2, treatment completion, 6-8 76months post-treatment	Whole blood (frozen)	Culture (NR)<br>Smear microscopy (NR)<br>GeneXpert (MTB/RIF)
	Sivakumaran (2020)	198 gene set profiled using dual-colour-Reverse-Transcriptase-Multiplex-Ligation-dependent-Probe-Amplification (dc-RT MLPA) platform	(74)	India	0, M1, M2, M6	Whole blood (frozen)	Liquid culture (MGIT)<br>Smear microscopy (FM)<br>Clinical outcome
	Warsinske (2018)	3-gene signature (*GBP5*, *DUSP3* and *KLF2*)	(75)	South Africa	0, W1, W4, W24	Whole blood (NR)	Liquid culture (MGIT)<br>PET-CT
Vascular endothelial growth factor (VEGF)	Djoba Siawaya (2009)	VEGF – Lincoplex human cytokine 29-plex assays (Millipore)	(34)	South Africa	0, W1, W5, W13, W26	Plasma (frozen)	Liquid culture (BACTEC 460T)<br>Smear microscopy (NR)
	Ferrian (2017)	VEGF-A on a multiplex (ProcartaPlex Human kits, eBioscience) read on a Bioplex 200 (BioRad)	(22)	South Africa	0, M2, M4, M6	Plasma (frozen)	Smear microscopy (NR)<br>Liquid culture (MGIT 960)<br>Line probe assay (Hain)
	Kumar (2016)	VEGF-A, VEGF-C, VEGF-R1, VEGF-R2 and VEGF-R3 using the Duoset ELISA Development System (R&D Systems)	(76)	India	0, M6	Platelet-poor plasma (fresh)	Culture (NR)
	Riou (2012)	VEGF – Multiplex bead array (Bulletin# 10014905 Bio-Rad) on a luminometer (Luminex)	(35)	South Africa	0, W2, W4, W8, W12, W26, W52, W78	Plasma (frozen)	Solid culture (LJ)<br>Liquid culture (MGIT)<br>Smear microscopy (FM)
Vitamin B12	Gebremicael (2019)	e601 module for Vitamin B12 on the cobas6000 analyser (Roche Diagnostics Corporation)	(47)	Ethiopia	0, M6	Serum (frozen)	Clinical outcome<br>Smear microscopy (ZN)
White blood cell (WBC) count	Chedid (2020)	Bangladesh: Callatac ES Automatec Hematology Analyzer, MEK-7300 (Nihon Kohden)<br>Georgia: XT-20001 (Sysmex)<br>Lebanon: CELL-DYN Ruby (Abbot<br>Madagascar: XT2100i and XN1000 (Sysmex)<br>Paraguay: XT-20001 (Sysmex)	(77)	Bangladesh, Georgia, Lebanon, Madagascar, Paraguay	0, M2, EOT, EOT+2M	Whole blood (NR)	Solid culture (LJ)<br>Liquid culture (MGIT)<br>Smear microscopy (ZN)<br>Smear microscopy (FM)

Table S6: Full list of studies and references included in the quantitative fold-change analyses

	Week 8 fold-change		Weekly fold-change (meta-regression)	
**Biomarker**	**Author (Year)***	**Ref**	**Author (Year)**	**Ref**
**CRP**	Almeida (2009)	(20)	Almeida (2009)	(20)
	Ferrian (2017)	(22)	Ferrian (2017)	(22)
	Jayakumar (2015)	(24)	Francisco (2017)	(23)
	Khalil (2020)	(25)	Jayakumar (2015)	(24)
	Miranda (2017)	(27)	Khalil (2020)	(25)
			Miranda (2017)	(27)
			De Moraes (2014)	
**IL-6**	Chowdhury (2014)	(42)	Chowdhury (2014)	(42)
	Feng (2020)	(51)	Djoba Siawaya (2009)	(34)
	Luo (2018)	(49)	Feng (2020)	(51)
	Mvungi (2019)	(46)	Luo (2018)	(49)
			Mvungi (2019)	(46)
**IP-10**	Chen (2011)	(40)	Chen (2011)	(40)
	Ferrian (2017)	(22)	Djoba Siawaya (2009)	(34)
	Jayakumar (2015)	(24)	Ferrian (2017)	(22)
	Kim (2018)	(58)	Francisco (2017)	(23)
			Garcia-Basteiro (2017)	(55)
			Hong (2013)	(56)
			Jayakumar (2015)	(24)
			Kim (2018)	(58)
			Riou (2012)	(35)
**TNF-α**	Chowdhury (2014)	(42)	Chowdhury (2014)	(42)
	Luo (2018)	(49)	Djoba Siawaya (2009)	(34)
	Mvungi (2019)	(46)	Luo (2018)	(49)
	Nie (2020)	(44)	Mvungi (2019)	(46)
			Nie (2020)	(44)
			Zhu (2015)	(54)

* Only papers that included week 8 data were included in this analysis.

Figure S7: Fold-changes plots for individual studies of CRP, IP-10, IL-6 and TNF-α

CRP

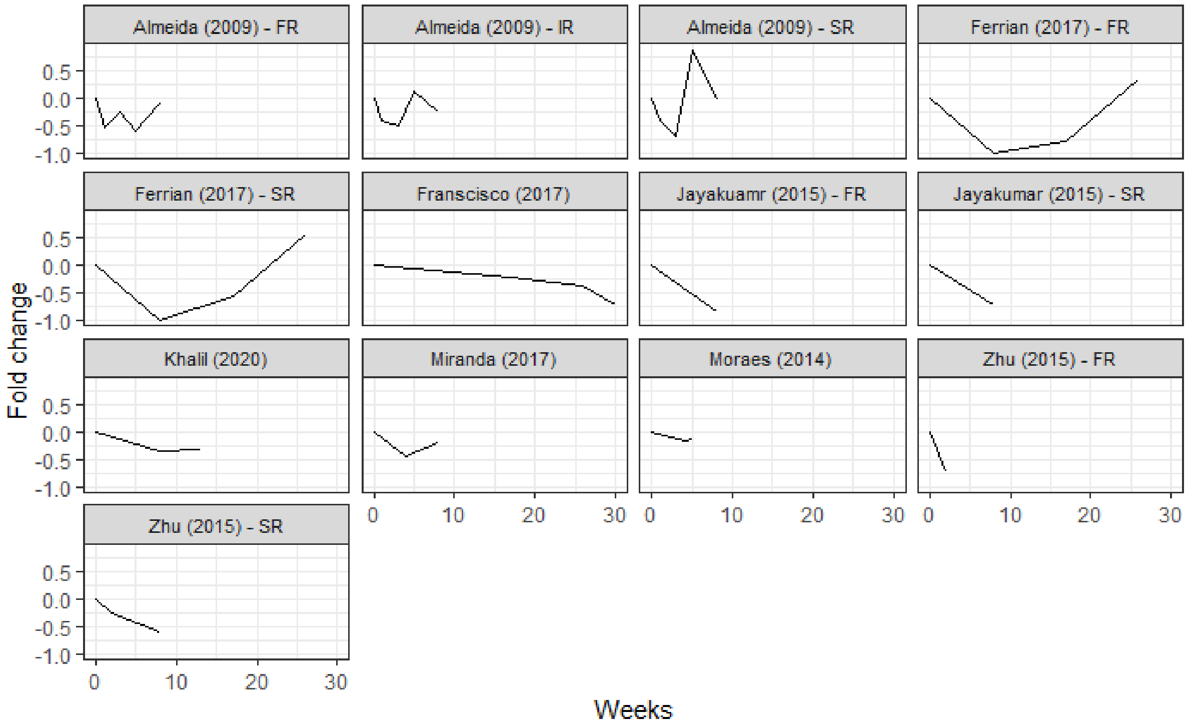

Figure S7.1: CRP fold change among TB patients on treatment across different studies. Fold change is relative to the previous time point reported in the study. FR – fast responders; IR – intermediate responders, SR – slow responders.

IL-6

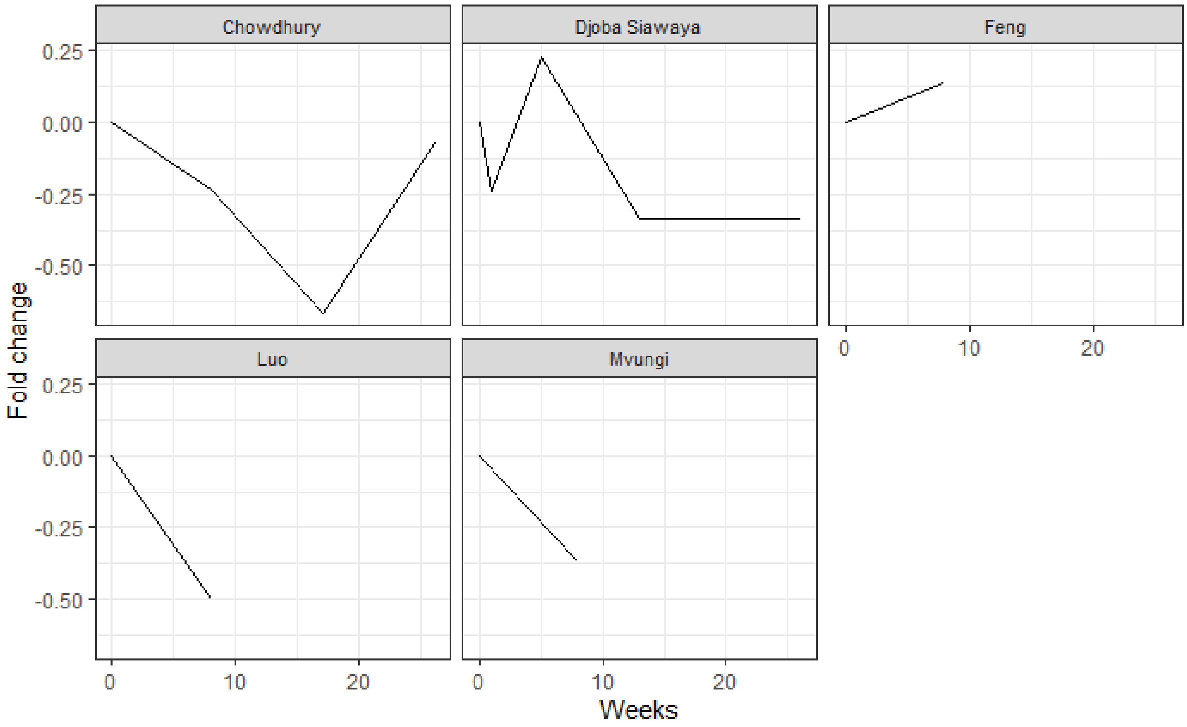

Figure S7.2: IL-6 change among TB patients on treatment across different studies. Fold change is relative to the previous time point reported in the study.

IP-10

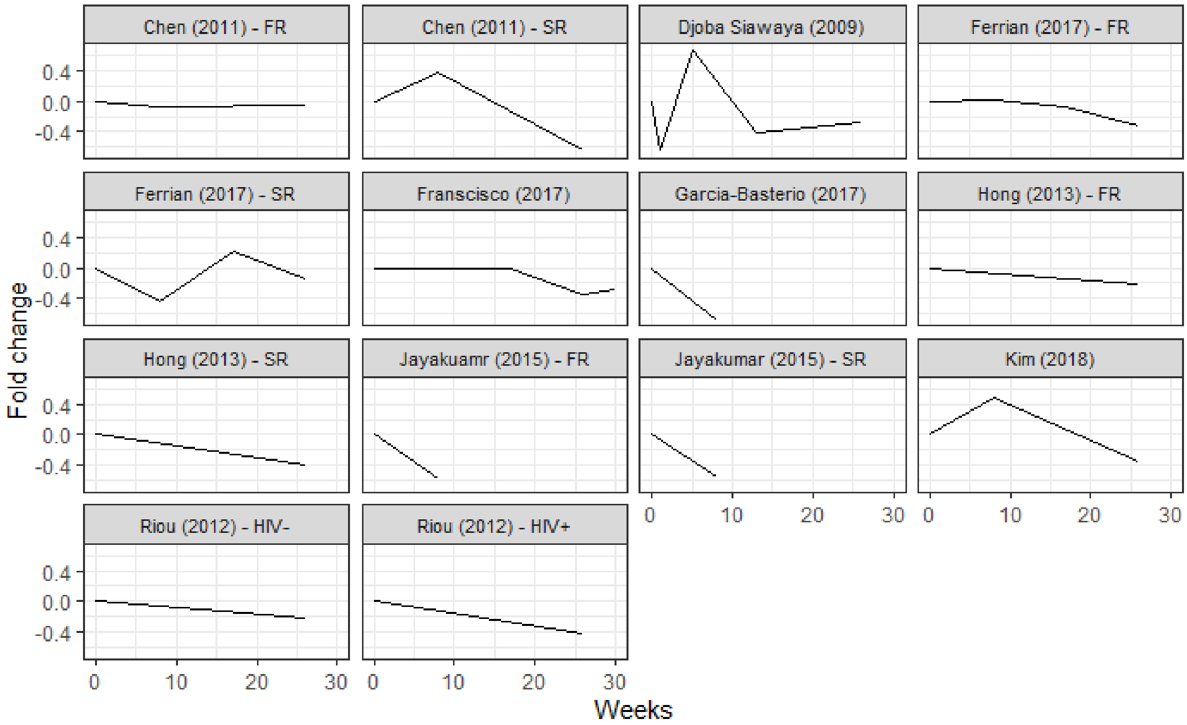

Figure S7.3: IP-10 fold change among TB patients on treatment across different studies. Fold change is relative to the previous time point reported in the study. FR – fast responders; SR – slow responders.

TNF-α

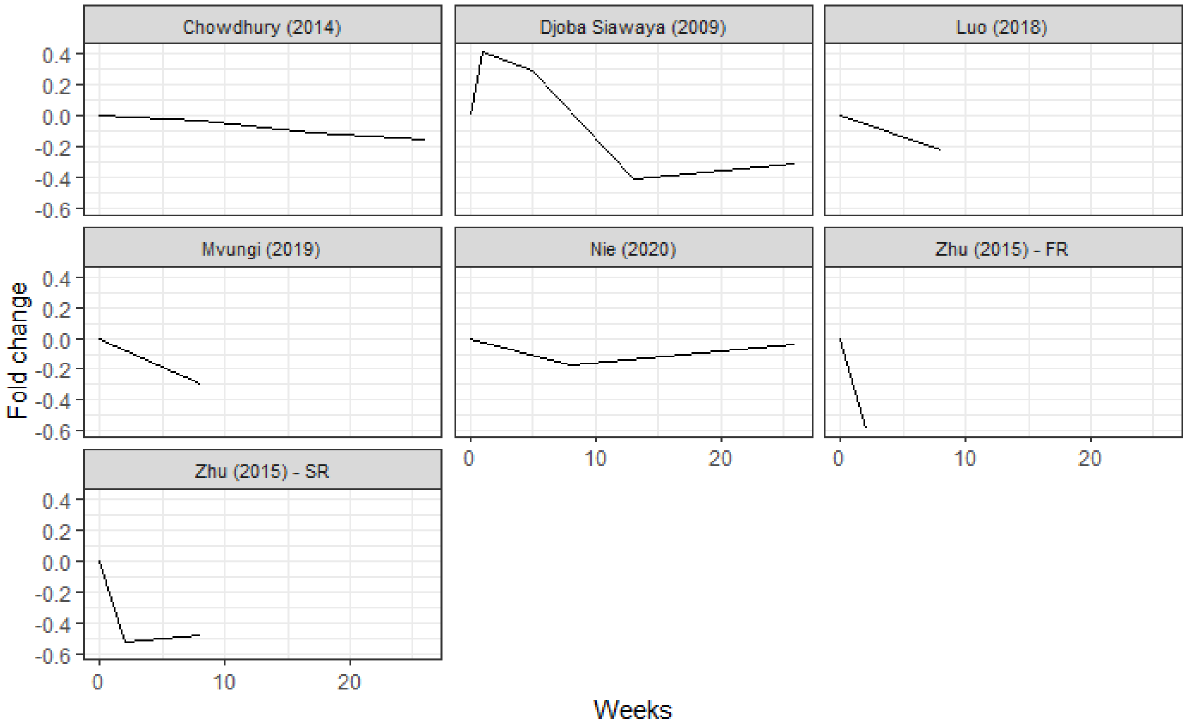

Figure S7.4: TNF-α fold change among TB patients on treatment across different studies. Fold change is relative to the previous time point reported in the study. FR – fast responders; SR – slow responders.

Table S8: Sensitivity analysis for the weekly fold-change meta-regression with varying correlation coefficient (ρ) parameter

	Correlation coefficient (ρ) (95% CI)			
	**0**	**0.25**	**0.5**	**0.75**
**CRP**	-0.546 (-0.702, -0.390)	-0.547 (-0.705, -0.389)	-0.539 (-0.702, -0.375)	-0.558 (-0.714, -0.401)
**IL-6**	-0.317 (-0.595, -0.038)	-0.313 (-0.596, -0.031)	-0.310 (-0.595, -0.025)	-0.306 (-0.592, -0.019)
**IP-10**	-0.357 (-0.497, -0.218)	-0.361 (-0.495, -0.227)	-0.362 (-0.490, -0.234)	-0.367 (-0.494, -0.241)
**TNF-α**	-0.119 (-0.171, -0.067)	-0.150 (-0.248, -0.053)	-0.177 (-0.313, -0.040)	-0.149 (-0.227, -0.071)

Table S9: Meta-analysis of outcome is the fold-change from previous recorded timepoint sensitivity analysis for CRP (excluding Ferrian et al.)

Biomarker	Estimate (95% CI)
CRP[1]	-0.539 (-0.702, -0.375)
CRP[2]	-0.453 (-0.594, -0.313)

[1]Including Ferrian et al.
[2]Excluding Ferrian et al.

Table S10: Meta-regression where the outcome is the fold-change from baseline and the predictor is treatment duration.

Biomarker	Estimate (95% CI)
CRP[1]	-0.0001 (-0.0002, 0.0001)
CRP[2]	-0.0518 (-0.0552, -0.0484)
IL-6	-0.0158 (-0.0160, -0.0156)
IP-10	-0.0320 (-0.0354, -0.0285)
TNF-α	-0.0094 (-0.0137, -0.0051)

Supplementary references

1. Hai HT, Vinh DN, Thu DDA, Hanh NT, Phu NH, Srinivasan V, et al. Comparison of the Mycobacterium tuberculosis molecular bacterial load assay, microscopy and GeneXpert versus liquid culture for viable bacterial load quantification before and after starting pulmonary tuberculosis treatment. Tuberculosis. 2019 Dec 1;119:101864.
2. Honeyborne I, McHugh TD, Phillips PPJ, Bannoo S, Bateson A, Carroll N, et al. Molecular bacterial load assay, a culture-free biomarker for rapid and accurate quantification of sputum Mycobacterium tuberculosis bacillary load during treatment. J Clin Microbiol [Internet]. 2011 Nov [cited 2021 Jun 20];49(11):3905–11. Available from: https://journals.asm.org/journal/jcm
3. Honeyborne I, Mtafya B, Phillips PPJ, Hoelscher M, Ntinginya EN, Kohlenberg A, et al. The molecular bacterial load assay replaces solid culture for measuring early bactericidal response to antituberculosis treatment. J Clin Microbiol [Internet]. 2014 [cited 2021 Jun 20];52(8):3064–7. Available from: https://journals.asm.org/journal/jcm
4. Sabiiti W, Azam K, Farmer ECW, Kuchaka D, Mtafya B, Bowness R, et al. Tuberculosis bacillary load, an early marker of disease severity: The utility of tuberculosis Molecular Bacterial Load Assay. Thorax [Internet]. 2020 Jul 1 [cited 2021 Jun 21];75(7):606–8. Available from: https://www.
5. Yan L, Xiao H, Zhang Q. Using simultaneous amplification and testing method for evaluating the treatment outcome of pulmonary tuberculosis. BMC Infect Dis [Internet]. 2018 Oct 11 [cited 2021 Jun 21];18(1):1–7. Available from: https://doi.org/10.1186/s12879-018-3424-y
6. Atahan E, Saribas S, Demirci M, Babalık A, Akkus S, Balıkcı A, et al. Evaluating the effectiveness of anti-tuberculosis treatment by detecting Mycobacterium tuberculosis 85B messenger RNA expression in sputum. J Infect Public Health. 2020 Oct 1;13(10):1490–4.
7. Montenegro RA, Guarines KM, Montenegro LML, Lira LAS, Falcão J, Melo FL, et al. Assessment of messenger RNA (mRNA) of Mycobacterium tuberculosis as a marker of cure in patients with pulmonary tuberculosis. J Appl Microbiol [Internet]. 2014 Jul 1 [cited 2021 Jun 21];117(1):266–72. Available from: http://www.ncbi.nlm.nih.
8. Mdivani N, Li H, Akhalaia M, Gegia M, Goginashvili L, Kernodle DS, et al. Monitoring therapeutic efficacy by real-time detection of Mycobacterium tuberculosis mRNA in sputum. Clin Chem [Internet]. 2009 Sep 1 [cited 2021 Jun 21];55(9):1694–700. Available from: http://www.clinchem.org/
9. Singh UB, Rana T, Kaushik A, Porwal C, Makkar N. Day zero quantitative mRNA analysis as a prognostic marker in pulmonary tuberculosis category II patients on treatment. Clin Microbiol Infect [Internet]. 2012 Nov 1 [cited 2021 Jun 21];18(11):E473–81. Available from: http://www.clinicalmicrobiologyandinfection.com/article/S1198743X14607606/fulltext
10. Altunoglu E, Erdenen F, Gelisgen R, Kar O, Korkmaz G, Muderrisoglu C, et al. Serum Adenosine Deaminase Activity and Neopterin Levels during Therapy in Patients with Pulmonary Tuberculosis and Community-Acquired Pneumonia. Istanbul Med J. 2014 Jun 16;15(2):78–82.
11. Ige O, Edem VF, Arinola OG. Plasma Adenosine Deaminase Enzyme Reduces with Treatment of Pulmonary Tuberculosis in Nigerian Patients: Indication for Diagnosis and Treatment Monitoring. Niger J Physiol Sci [Internet]. 2016 [cited 2021 Jun 20];31:53. Available from: www.njps.com.ng
12. De Moraes ML, Ramalho DMDP, Delogo KN, Miranda PFC, Mesquita EDD, De Melo Guedes De Oliveira HM, et al. Association of serum levels of iron, copper, and zinc, and inflammatory markers with bacteriological sputum conversion during

tuberculosis treatment. Biol Trace Elem Res [Internet]. 2014 Jun 24 [cited 2021 Jun 21];160(2):176–84. Available from: https://link.springer.com/article/10.1007/s12011-014-0046-0

13. Jiang TT, Shi LY, Chen J, Wei LL, Li M, Hu YT, et al. Screening and identification of potential protein biomarkers for evaluating the efficacy of intensive therapy in pulmonary tuberculosis. Biochem Biophys Res Commun. 2018 Sep 18;503(4):2263–70.

14. Kumar NP, Velayutham B, Nair D, Babu S. Angiopoietins as biomarkers of disease severity and bacterial burden in pulmonary tuberculosis. Int J Tuberc Lung Dis. 2017 Jan 1;21(1).

15. Mendy J, Togun T, Owolabi O, Donkor S, Ota MOC, Sutherland JS. C-reactive protein, Neopterin and Beta2 microglobulin levels pre and post TB treatment in The Gambia. BMC Infect Dis [Internet]. 2016 Mar 8 [cited 2021 Jun 21];16(1):1–6. Available from: https://bmcinfectdis.biomedcentral.com/articles/10.1186/s12879-016-1447-9

16. Kim ES, Park KU, Song J, Lim HJ, Cho YJ, Yoon H, et al. The clinical significance of CA-125 in pulmonary tuberculosis. Tuberculosis. 2013 Mar 1;93(2):222–6.

17. Sahin F, Yildiz P. Serum CA-125: Biomarker of pulmonary tuberculosis activity and evaluation of response to treatment. Clin Investig Med. 2012;35(4).

18. Said AF, Mohamed BI, El-Sharkawy E, Al-Sherif M. Role of cancer antigen 125 in active pulmonary tuberculosis. Egypt J Chest Dis Tuberc. 2013 Jul 1;62(3):419–24.

19. Lee GO, Comina G, Hernandez-Cordova G, Naik N, Gayoso O, Ticona E, et al. Cough dynamics in adults receiving tuberculosis treatment. PLoS One [Internet]. 2020 Jun 1 [cited 2021 Jun 21];15(6):e0231167. Available from: https://doi.org/10.1371/journal.pone.0231167

20. Almeida MLD, Barbieri MA, Gurgel RQ, Abdurrahman ST, Baba UA, Hart CA, et al. α1-acid glycoprotein and α1-antitrypsin as early markers of treatment response in patients receiving the intensive phase of tuberculosis therapy. Trans R Soc Trop Med Hyg [Internet]. 2009 Jun 1 [cited 2021 Jun 20];103(6):575–80. Available from: https://academic.oup.com/trstmh/article/103/6/575/1933610

21. Djoba Siawaya JF, Bapela NB, Ronacher K, Veenstra H, Kidd M, Gie R, et al. Immune parameters as markers of tuberculosis extent of disease and early prediction of anti-tuberculosis chemotherapy response. J Infect [Internet]. 2008 May 1 [cited 2021 Jun 21];56(5):340–7. Available from: http://www.journalofinfection.com/article/S0163445308000844/fulltext

22. Ferrian S, Manca C, Lubbe S, Conradie F, Ismail N, Kaplan G, et al. A combination of baseline plasma immune markers can predict therapeutic response in multidrug resistant tuberculosis. PLoS One [Internet]. 2017 May 1 [cited 2021 Jun 20];12(5):e0176660. Available from: https://doi.org/10.1371/journal.pone.0176660

23. Francisco NM, Fang YM, Ding L, Feng S, Yang Y, Wu M, et al. Diagnostic accuracy of a selected signature gene set that discriminates active pulmonary tuberculosis and other pulmonary diseases. J Infect. 2017 Dec;75(6):499–510.

24. Jayakumar A, Vittinghoff E, Segal MR, MacKenzie WR, Johnson JL, Gitta P, et al. Serum biomarkers of treatment response within a randomized clinical trial for pulmonary tuberculosis. Tuberculosis. 2015 Jul 1;95(4):415–20.

25. Khalil M, Halim H., Abdelazeem M. C-reactive protein versus erythrocyte sedimentation rate in monitoring multidrug-resistant tuberculosis. Egypt J Chest Dis Tuberc [Internet]. 2020 [cited 2021 Jun 20];69(3):458. Available from: http://www.ejcdt.eg.net/article.asp?issn=0422-7638;year=2020;volume=69;issue=3;spage=458;epage=465;aulast=Khalil

26. Mesquita EDD, Gil-Santana L, Ramalho D, Tonomura E, Silva EC, Oliveira MM, et al. Associations between systemic inflammation, mycobacterial loads in sputum and

radiological improvement after treatment initiation in pulmonary TB patients from Brazil: A prospective cohort study. BMC Infect Dis [Internet]. 2016 Aug 5 [cited 2021 Jun 21];16(1):1–12. Available from: https://bmcinfectdis.biomedcentral.com/articles/10.1186/s12879-016-1736-3

27. Miranda P, Gil-Santana L, Oliveira MG, Mesquita EDD, Silva E, Rauwerdink A, et al. Sustained elevated levels of C-reactive protein and ferritin in pulmonary tuberculosis patients remaining culture positive upon treatment initiation. PLoS One [Internet]. 2017 Apr 1 [cited 2021 Jun 21];12(4):e0175278. Available from: https://doi.org/10.1371/journal.pone.0175278

28. Sigal GB, Segal MR, Mathew A, Jarlsberg L, Wang M, Barbero S, et al. Biomarkers of Tuberculosis Severity and Treatment Effect: A Directed Screen of 70 Host Markers in a Randomized Clinical Trial. EBioMedicine [Internet]. 2017 Nov 1 [cited 2021 Jun 21];25:112–21. Available from: http://creativecommons.org/licenses/by/4.0/

29. Chung W, Lee K, Jung Y, Kim Y, Park J, Sheen S, et al. Serum CXCR3 ligands as biomarkers for the diagnosis and treatment monitoring of tuberculosis. Int J Tuberc Lung Dis. 2015 Dec 1;19(12):1476–84.

30. Chung WY, Yoon D, Lee KS, Jung YJ, Kim YS, Sheen SS, et al. The Usefulness of Serum CXCR3 Ligands for Evaluating the Early Treatment Response in Tuberculosis. Med (United States) [Internet]. 2016 Apr 1 [cited 2021 Jun 20];95(17). Available from: https://journals.lww.com/md-journal/Fulltext/2016/04260/The_Usefulness_of_Serum_CXCR3_Ligands_for.57.aspx

31. Lee MR, Tsai CJ, Wang WJ, Chuang TY, Yang CM, Chang LY, et al. Plasma biomarkers can predict treatment response in tuberculosis patients: A prospective observational study. Med (United States) [Internet]. 2015 [cited 2021 Jun 21];94(39):e1628. Available from: https://journals.lww.com/md-journal/Fulltext/2015/09050/Plasma_Biomarkers_Can_Predict_Treatment_Response.48.aspx

32. Wang X, Tang J, Wang R, Chen C, Tan S, Yu F, et al. Sputum endothelin-1 level is associated with active pulmonary tuberculosis and effectiveness of anti-tuberculosis chemotherapy. Exp Ther Med [Internet]. 2016 Mar 1 [cited 2021 Jun 21];11(3):1104–8. Available from: http://www.spandidos-publications.com/10.3892/etm.2016.2980/abstract

33. Choi R, Kim K, Kim MJ, Kim SY, Kwon OJ, Jeon K, et al. Serum inflammatory profiles in pulmonary tuberculosis and their association with treatment response. J Proteomics. 2016 Oct 21;149:23–30.

34. Djoba Siawaya JF, Beyers N, Van Helden P, Walzl G. Differential cytokine secretion and early treatment response in patients with pulmonary tuberculosis. Clin Exp Immunol [Internet]. 2009 Apr 1 [cited 2021 Jun 20];156(1):69–77. Available from: https://onlinelibrary.wiley.com/doi/full/10.1111/j.1365-2249.2009.03875.x

35. Riou C, Perez Peixoto B, Roberts L, Ronacher K, Walzl G, Manca C, et al. Effect of standard tuberculosis treatment on plasma cytokine levels in patients with active pulmonary tuberculosis. PLoS One [Internet]. 2012 May 14 [cited 2021 Jun 21];7(5):e36886. Available from: www.plosone.org

36. Ralph AP, Yeo TW, Salome CM, Waramori G, Pontororing GJ, Kenangalem E, et al. Impaired pulmonary nitric oxide bioavailability in pulmonary tuberculosis: Association with disease severity and delayed mycobacterial clearance with treatment. J Infect Dis [Internet]. 2013 Aug 15 [cited 2021 Jun 21];208(4):616–26. Available from: https://academic.oup.com/jid/article/208/4/616/920444

37. Zetola NM, Modongo C, Matsiri O, Tamuhla T, Mbongwe B, Matlhagela K, et al. Diagnosis of pulmonary tuberculosis and assessment of treatment response through analyses of volatile compound patterns in exhaled breath samples. J Infect [Internet]. 2017 Apr 1 [cited 2021 Jun 21];74(4):367–76. Available from:

http://www.journalofinfection.com/article/S016344531630336X/fulltext

38. Andrade BB, Pavan Kumar N, Mayer-Barber KD, Barber DL, Sridhar R, Rekha VVB, et al. Plasma Heme Oxygenase-1 Levels Distinguish Latent or Successfully Treated Human Tuberculosis from Active Disease. PLoS One [Internet]. 2013 May 6 [cited 2021 Jun 20];8(5):e62618. Available from: www.plosone.org

39. Rockwood N, Costa DL, Amaral EP, Du Bruyn E, Kubler A, Gil-Santana L, et al. Mycobacterium tuberculosis induction of heme oxygenase-1 expression is dependent on oxidative stress and reflects treatment outcomes. Front Immunol [Internet]. 2017 May 12 [cited 2021 Jun 21];8(MAY):542. Available from: www.frontiersin.org

40. Chen YC, Chin CH, Liu SF, Wu CC, Tsen CC, Wang YH, et al. Prognostic values of serum IP-10 and IL-17 in patients with pulmonary tuberculosis. Dis Markers. 2011;31(2):101–10.

41. Ehtesham NZ, Nasiruddin M, Alvi A, Kumar BK, Ahmed N, Peri S, et al. Treatment end point determinants for pulmonary tuberculosis: Human resistin as a surrogate biomarker. Tuberculosis. 2011 Jul 1;91(4):293–9.

42. Chowdhury IH, Ahmed AM, Choudhuri S, Sen A, Hazra A, Pal NK, et al. Alteration of serum inflammatory cytokines in active pulmonary tuberculosis following anti-tuberculosis drug therapy. Mol Immunol. 2014 Nov 1;62(1):159–68.

43. Siawaya JFD, Bapela NB, Ronacher K, Beyers N, Van Helden P, Walzl G. Differential Expression of Interleukin-4 (IL-4) and IL-4δ2 mRNA, but Not Transforming Growth Factor Beta (TGF-β), TGF-βRII, Foxp3, Gamma Interferon, T-bet, or GATA-3 mRNA, in Patients with Fast and Slow Responses to Antituberculosis Treatment. Clin Vaccine Immunol [Internet]. 2008 Aug [cited 2021 Jun 21];15(8):1165–70. Available from: https://journals.asm.org/journal/cvi

44. Nie W, Wang J, Jing W, Shi W, Wang Q, Huang X, et al. Value of serum cytokine biomarkers TNF-α, IL-4, sIL-2R and IFN-γ for use in monitoring bacterial load and anti-tuberculosis treatment progress. Cytokine X. 2020 Jun 1;2(2):100028.

45. Matsushita I, Hang NT Le, Hong LT, Tam DB, Lien LT, Thuong PH, et al. Dynamics of immune parameters during the treatment of active tuberculosis showing negative interferon gamma response at the time of diagnosis. Int J Infect Dis [Internet]. 2015 Nov 1 [cited 2021 Jun 21];40:39–44. Available from: http://dx.doi.org/10.1016/j.ijid.2015.09.021

46. Mvungi HC, Mbelele PM, Buza JJ, Mpagama SG, Sauli E. Blood cytokine responses to early secreted protein antigen-6/culture filtrate protein-10 tuberculosis antigens 2 months after antituberculosis treatment among patients with drug-susceptible pulmonary tuberculosis. Int J Mycobacteriology [Internet]. 2019 Jan 1 [cited 2021 Jun 21];8(1):53–9. Available from: https://www.ijmyco.org/article.asp?issn=2212-5531;year=2019;volume=8;issue=1;spage=53;epage=59;aulast=Mvungi

47. Gebremicael G, Alemayehu M, Sileshi M, Geto Z, Gebreegziabxier A, Tefera H, et al. The serum concentration of vitamin B12 as a biomarker of therapeutic response in tuberculosis patients with and without human immunodeficiency virus (HIV) infection. Int J Gen Med [Internet]. 2019 Sep 20 [cited 2021 Jun 20];12:353–61. Available from: http://doi.org/10.2147/IJGM.S218799

48. Anusiem CA, Okonkwo PO. The impact of treatment on the serum concentration of interleukin-1 beta in pulmonary tuberculosis. Am J Ther [Internet]. 2017 May 1 [cited 2021 Jun 20];24(3):e329–32. Available from: https://journals.lww.com/americantherapeutics/Fulltext/2017/05000/The_Impact_of_Treatment_on_the_Serum_Concentration.11.aspx

49. Luo X, Wu F, Ma J, Xiao H, Cui H. Immunological recovery in patients with pulmonary tuberculosis after intensive phase treatment. J Int Med Res [Internet]. 2018 Sep 1 [cited 2021 Jun 21];46(9):3539–51. Available from: https://us.sagepub.com/en-us/nam/open-access-at-sage

50. Alzubaidi LKA, Alyasiri NS, Mehdi LY. Study the level of interlukin-2 during and after treatment in sputum of Iraqi patients with pulmonary tuberculosis. Indian J Public Heal Res Dev. 2019 Sep 1;10(9):746–51.
51. Feng JY, Ho LI, Chuang FY, Pan SW, Chen YY, Tung CL, et al. Depression and recovery of IL-17A secretion in mitogen responses in patients with active tuberculosis-a prospective observational study. J Formos Med Assoc. 2021 Apr 1;120(4):1080–9.
52. Kumar NP, Banurekha V V., Nair D, Babu S. Diminished plasma levels of common γ-chain cytokines in pulmonary tuberculosis and reversal following treatment. PLoS One [Internet]. 2017 Apr 1 [cited 2021 Jun 20];12(4):e0176495. Available from: https://doi.org/10.1371/journal.pone.0176495
53. Zhao G, Luo X, Han X, Liu Z. Combining bioinformatics and biological detection to identify novel biomarkers for diagnosis and prognosis of pulmonary tuberculosis. Saudi Med J [Internet]. 2020 Apr 1 [cited 2021 Jun 21];41(4):351–60. Available from: http://orcid.org/0000-0002-5939-7140.www.smj.org.sa
54. Zhu Y, Jia H, Chen J, Cui G, Gao H, Wei Y, et al. Decreased Osteopontin Expression as a Reliable Prognostic Indicator of Improvement in Pulmonary Tuberculosis: Impact of the Level of Interferon-γ-Inducible Protein 10. Cell Physiol Biochem [Internet]. 2015 Nov 1 [cited 2021 Jun 21];37(5):1983–96. Available from: www.karger.com/cpb1983
55. García-Basteiro AL, Mambuque E, Den Hertog A, Saavedra B, Cuamba I, Oliveras L, et al. IP-10 Kinetics in the First Week of Therapy are Strongly Associated with Bacteriological Confirmation of Tuberculosis Diagnosis in HIV-Infected Patients. Sci Rep [Internet]. 2017 Dec 1 [cited 2021 Jun 20];7(1):1–8. Available from: www.nature.com/scientificreports/
56. Hong JY, Lee HJ, Kim SY, Chung KS, Kim EY, Jung JY, et al. Efficacy of IP-10 as a biomarker for monitoring tuberculosis treatment. J Infect [Internet]. 2014 Mar 1 [cited 2021 Jun 20];68(3):252–8. Available from: http://www.journalofinfection.com/article/S0163445313003368/fulltext
57. Kabeer BSA, Raja A, Raman B, Thangaraj S, Leportier M, Ippolito G, et al. IP-10 response to RD1 antigens might be a useful biomarker for monitoring tuberculosis therapy. BMC Infect Dis [Internet]. 2011 May 19 [cited 2021 Jun 20];11(1):1–9. Available from: http://www.biomedcentral.com/1471-2334/11/135
58. Kim SY, Kim J, Kim DR, Kang YA, Bong S, Lee J, et al. Urine IP-10 as a biomarker of therapeutic response in patients with active pulmonary tuberculosis. BMC Infect Dis [Internet]. 2018 May 29 [cited 2021 Jun 20];18(1):1–6. Available from: https://doi.org/10.1186/s12879-018-3144-3
59. Drain PK, Gounder L, Grobler A, Sahid F, Bassett I V., Moosa MYS. Urine lipoarabinomannan to monitor antituberculosis therapy response and predict mortality in an HIV-endemic region:A prospective cohort study. BMJ Open [Internet]. 2015 Apr 1 [cited 2021 Jun 20];5(4):e006833. Available from: http://bmjopen.bmj.com/
60. Kawasaki M, Echiverri C, Raymond L, Cadena E, Reside E, Gler MT, et al. Lipoarabinomannan in sputum to detect bacterial load and treatment response in patients with pulmonary tuberculosis: Analytic validation and evaluation in two cohorts. PLoS Med [Internet]. 2019 [cited 2021 May 17];16(4):e1002780. Available from: https://doi.org/10.1371/journal.pmed.1002780
61. Wood R, Racow K, Bekker LG, Middelkoop K, Vogt M, Kreiswirth BN, et al. Lipoarabinomannan in urine during tuberculosis treatment: Association with host and pathogen factors and mycobacteriuria. BMC Infect Dis [Internet]. 2012 Feb 27 [cited 2021 Jun 21];12(1):1–11. Available from: http://www.biomedcentral.com/1471-2334/12/47
62. Feruglio SL, Trøseid M, Damås JK, Kvale D, Dyrhol-Riise AM. Soluble Markers of the Toll-Like Receptor 4 Pathway Differentiate between Active and Latent

Tuberculosis and Are Associated with Treatment Responses. PLoS One [Internet]. 2013 Jul 16 [cited 2021 Jun 20];8(7):e69896. Available from: www.plosone.org

63. Ugarte-Gil CA, Elkington P, Gilman RH, Coronel J, Tezera LB, Bernabe-Ortiz A, et al. Induced Sputum MMP-1, -3 & -8 Concentrations during Treatment of Tuberculosis. PLoS One [Internet]. 2013 Apr 22 [cited 2021 Jun 21];8(4):e61333. Available from: www.plosone.org

64. Osawa T, Watanabe M, Morimoto K, Okumura M, Yoshiyama T, Ogata H, et al. Serum procalcitonin levels predict mortality risk in patients with pulmonary tuberculosis: A single-center prospective observational study. J Infect Dis [Internet]. 2020 Nov 15 [cited 2021 Jun 21];222(10):1651–4. Available from: https://academic.oup.com/jid/article/222/10/1651/5843442

65. Barry SE, Ellis M, Yang YR, Guan G, Wang X, Britton WJ, et al. Identification of a plasma microRNA profile in untreated pulmonary tuberculosis patients that is modulated by anti-mycobacterial therapy. J Infect [Internet]. 2018 Oct 1 [cited 2021 Jun 20];77(4):341–8. Available from: http://www.journalofinfection.com/article/S0163445318301221/fulltext

66. Honeyborne I, Lipman MC, Eckold C, Evangelopoulos D, Gillespie SH, Pym A, et al. Effective anti-tuberculosis therapy correlates with plasma small RNA [Internet]. Vol. 45, European Respiratory Journal. European Respiratory Society; 2015 [cited 2021 Jun 20]. p. 1741–4. Available from: http://ow.ly/IxOaD

67. Rabna P, Andersen A, Wejse C, Oliveira I, Gomes VF, Haaland MB, et al. Utility of the Plasma Level of suPAR in Monitoring Risk of Mortality during TB Treatment. PLoS One [Internet]. 2012 Aug 28 [cited 2021 Jun 21];7(8):e43933. Available from: www.plosone.org

68. Mardining Raras TY, Noor Chozin I. The Soluble Plasminogen Activator Receptor as a Biomarker on Monitoring the Therapy Progress of Pulmonary TB-AFB(+) Patients . Tuberc Res Treat. 2010;2010:1–6.

69. Ronacher K, Chegou NN, Kleynhans L, Djoba Siawaya JF, du Plessis N, Loxton AG, et al. Distinct serum biosignatures are associated with different tuberculosis treatment outcomes. Tuberculosis. 2019 Sep 1;118:101859.

70. Bloom CI, Graham CM, Berry MPR, Wilkinson KA, Oni T, Rozakeas F, et al. Detectable Changes in The Blood Transcriptome Are Present after Two Weeks of Antituberculosis Therapy. PLoS One [Internet]. 2012 Oct 2 [cited 2021 May 4];7(10):e46191. Available from: www.plosone.org

71. Darboe F, Mbandi SK, Naidoo K, Yende-Zuma N, Lewis L, Thompson EG, et al. Detection of tuberculosis recurrence, diagnosis and treatment response by a blood transcriptomic risk signature in HIV-infected persons on antiretroviral therapy. Front Microbiol [Internet]. 2019 Jun 26 [cited 2021 Jun 22];10(JUN):1441. Available from: www.frontiersin.org

72. Gebremicael G, Kassa D, Alemayehu Y, Gebreegziaxier A, Kassahun Y, van Baarle D, et al. Gene expression profiles classifying clinical stages of tuberculosis and monitoring treatment responses in Ethiopian HIV-negative and HIV-positive cohorts. PLoS One [Internet]. 2019 Dec 1 [cited 2021 Jun 20];14(12):e0226137. Available from: https://doi.org/10.1371/journal.pone.0226137

73. Penn-Nicholson A, Mbandi SK, Thompson E, Mendelsohn SC, Suliman S, Chegou NN, et al. RISK6, a 6-gene transcriptomic signature of TB disease risk, diagnosis and treatment response. Sci Rep [Internet]. 2020 Dec 1 [cited 2021 May 4];10(1):1–21. Available from: https://doi.org/10.1038/s41598-020-65043-8

74. Sivakumaran D, Jenum S, Vaz M, Selvam S, Ottenhoff THM, Haks MC, et al. Combining host-derived biomarkers with patient characteristics improves signature performance in predicting tuberculosis treatment outcomes. Commun Biol [Internet]. 2020 Dec 1 [cited 2021 Jun 21];3(1):1–10. Available from:

https://doi.org/10.1038/s42003-020-1087-x
75. Warsinske HC, Rao AM, Moreira FMF, Santos PCP, Liu AB, Scott M, et al. Assessment of Validity of a Blood-Based 3-Gene Signature Score for Progression and Diagnosis of Tuberculosis, Disease Severity, and Treatment Response. JAMA Netw open. 2018;1(6):e183779.
76. Kumar NP, Banurekha V V., Nair D, Babu S. Circulating angiogenic factors as biomarkers of disease severity and bacterial burden in pulmonary tuberculosis. PLoS One [Internet]. 2016 Jan 4 [cited 2021 Jun 21];11(1):e0146318. Available from: https://journals.plos.org/plosone/article?id=10.1371/journal.pone.0146318
77. Chedid C, Kokhreidze E, Tukvadze N, Banu S, Uddin MKM, Biswas S, et al. Association of baseline white blood cell counts with tuberculosis treatment outcome: a prospective multicentered cohort study. Int J Infect Dis [Internet]. 2020 Nov 1 [cited 2021 Jun 20];100:199–206. Available from: http://creativecommons.org/licenses/by/4.0/
